# *Campylobacter jejuni* Type VI Secretion System: Roles in Adaptation to Deoxycholic Acid, Host Cell Adherence, Invasion, and *In Vivo* Colonization

**DOI:** 10.1371/journal.pone.0042842

**Published:** 2012-08-27

**Authors:** Kvin Lertpiriyapong, Eric R. Gamazon, Yan Feng, Danny S. Park, Jassia Pang, Georgina Botka, Michelle E. Graffam, Zhongming Ge, James G. Fox

**Affiliations:** 1 Division of Comparative Medicine, Massachusetts Institute of Technology, Cambridge, Massachusetts, United States of America; 2 Section of Genetic Medicine, Department of Medicine, The University of Chicago, Chicago, Illinois, United States of America; 3 Department of Computer Science, Columbia University, New York, New York, United States of America; Charité-University Medicine Berlin, Germany

## Abstract

The recently identified type VI secretion system (T6SS) of proteobacteria has been shown to promote pathogenicity, competitive advantage over competing microorganisms, and adaptation to environmental perturbation. By detailed phenotypic characterization of loss-of-function mutants, *in silico*, *in vitro* and *in vivo* analyses, we provide evidence that the enteric pathogen, *Campylobacter jejuni*, possesses a functional T6SS and that the secretion system exerts pleiotropic effects on two crucial processes – survival in a bile salt, deoxycholic acid (DCA), and host cell adherence and invasion. The expression of T6SS during initial exposure to the upper range of physiological levels of DCA (0.075%–0.2%) was detrimental to *C. jejuni* proliferation, whereas down-regulation or inactivation of T6SS enabled *C. jejuni* to resist this effect. The *C. jejuni* multidrug efflux transporter gene, *cmeA*, was significantly up-regulated during the initial exposure to DCA in the wild type *C. jejuni* relative to the T6SS-deficient strains, suggesting that inhibition of proliferation is the consequence of T6SS-mediated DCA influx. A sequential modulation of the efflux transporter activity and the T6SS represents, in part, an adaptive mechanism for *C. jejuni* to overcome this inhibitory effect, thereby ensuring its survival. *C. jejuni* T6SS plays important roles in host cell adhesion and invasion as T6SS inactivation resulted in a reduction of adherence to and invasion of *in vitro* cell lines, while over-expression of a *hemolysin co-regulated protein*, which encodes a secreted T6SS component, greatly enhanced these processes. When inoculated into B6.129P2-*IL-10^tm1Cgn^* mice, the T6SS-deficient *C. jejuni* strains did not effectively establish persistent colonization, indicating that T6SS contributes to colonization *in vivo.* Taken together, our data demonstrate the importance of bacterial T6SS in host cell adhesion, invasion, colonization and, for the first time to our knowledge, adaptation to DCA, providing new insights into the role of T6SS in *C. jejuni* pathogenesis.

## Introduction

Of the diverse arsenals that have been developed by proteobacteria, the recently identified type VI secretion system (T6SS) has proven to be functionally versatile [1], [2]. Not only does it promote pathogenicity, symbiotic relationships and a selective advantage among competing microbes, it also plays a pivotal role in the adaptation to environmental perturbation [1]. Since its discovery, T6SS gene clusters have been identified in over 25% of the known proteobacteria [3]. These bacteria have a remarkable range of colonization niches, including the soil, the marine environment, plants, invertebrates, vertebrates, and mammals [4]–[6]. T6SS consists of at least thirteen components that form injectisomes structurally resembling an upside-down *Escherichia coli* bacteriophage T4 [3], [7], [8]. In *Salmonella enterica* serotypes Gallinarum and Enteritidis, the T6SS enhances the ability of the bacteria to colonize the gastrointestinal tract of chickens [9]. In *Helicobacter hepaticus*, the T6SS enables the bacteria to establish non-pathogenic, symbiotic relationship in the gastrointestinal tract of mice by modulating the bacteria's virulence potential and the mouse's immune responses [10]. The pathogenic properties of T6SS have also been demonstrated in human pathogens, such as *Pseudomonas aeruginosa* and *Francisella tularensis* [11], [12]. Moreover, T6SS has been implicated in adaptation to high osmolarity and low temperature [13], quorum and stress sensing [11], [14], [15], bacterial growth and motility [16], biofilm formation [17], and destruction of competing bacteria and protozoa through secretion of toxic effector proteins [18], [19].

*Campylobacter jejuni*, a gram-negative and spiral-shaped microaerophilic bacterium, is a leading cause of human food-borne enterocolitis worldwide with approximately 400 million cases diagnosed each year [20]. Additionally, *C. jejuni* infection can result in debilitating extraintestinal complications including immune mediated polyarthritis and paralyzing autoimmune neuropathies– Guillain-Barré syndrome and Miller Fisher syndrome [21]. To date, over 10,000 isolates of *C. jejuni*, showing a considerable degree of genetic variability and pathogenic potential, have been identified and categorized (http://pubmlst.org/campylobacter). Analyses of 17 completed and partially completed genomic sequences of *Campylobacter* deposited in the European Bioinformatic Institute (EBI) or the National Center for Biotechnology Information (NCBI) performed by our laboratory revealed that select strains of *C. jejuni* and *Campylobacter* spp. contain unique clusters of T6SS core orthologs capable of forming a fully functional secretion apparatus. Proteins homologous to known components of T6SS were detected in *C. concisus*, indicating the presence of a functional T6SS in this bacterium [22]. Based on this finding and recognizing the known function of T6SS in other proteobacteria, we hypothesized that *C. jejuni* contains a functional T6SS and that the secretion system may provide the bacteria niche-specific adaptive capacities and/or enhance their pathogenic potential. To test this hypothesis, we created and characterized, using *in vitro* and *in vivo* analyses, a series of isogenic mutants that are defective in two highly conserved core T6SS orthologs– *icmF* (*intracellular multiplication F*) and *hcp* (*hemolysin co-regulated protein*) [3] –from a human clinical isolate, *C. jejuni* ATCC 43431. Hcp either forms a structural component similar to the gp27 component of the cell puncturing device of bacteriophage T4, or serves as a secreted effector protein that modulates host actin cytoskeleton rearrangement or cytokine production [7], [23]. IcmF is a structural transmembrane component required for secretion and assembly of Hcp and other T6SS constituents [3], [24].

## Results

### *C. jejuni* T6SS consists of a unique set of core and accessory components fully capable of secreting Hcp

**Figure 1:**
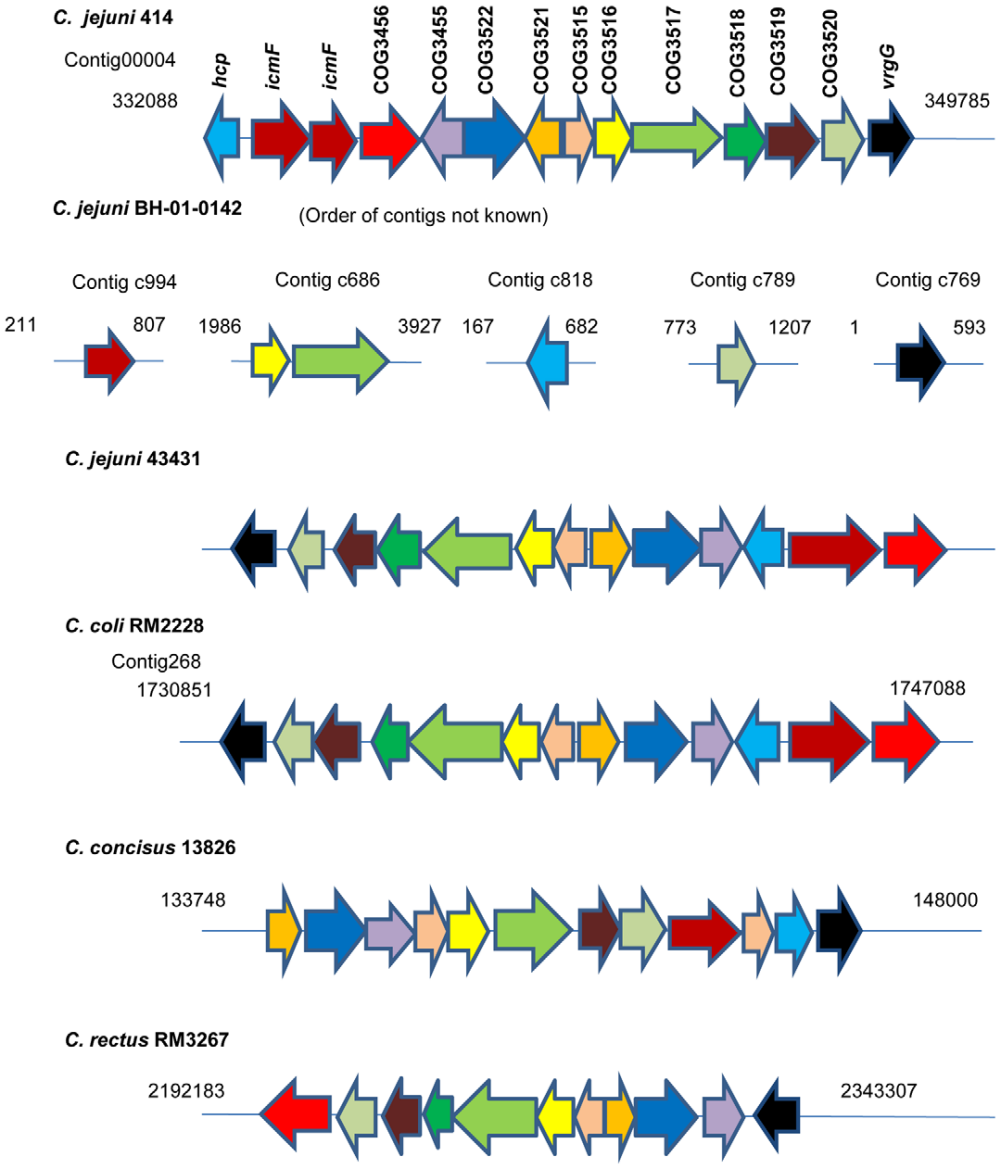
Gene organization of T6SS clusters in *Campylobacter* spp. Genes are plotted as arrows, starting from the beginning of the first gene to the end of the last gene, in order of their genomic positions. Gene sizes (defined as the distance between the annotated starts and ends) are represented by the relative length of the arrow. Arrow direction indicates the strand (i.e., forward arrows indicating genes on the positive strand and reverse arrows indicating genes on the negative strand). For each strain, genes are plotted using the color code indicated in *C. jejuni* 414.

Most known T6SSs of proteobacteria consist of at least 13 core genes [3], [7]. Our *in silico* analyses (see **Materials and Methods**) revealed that a similar cluster of 13 genes were present in 2 of 14 strains of *C. jejuni* analyzed (Figure 1) – *C. jejuni* ATCC 43431, which was isolated from a diarrheic human patient, as well as *C. jejuni* 414, isolated from bank voles [25], [26]. Furthermore, 6 T6SS orthologs were identified in the partially-sequenced genome of a human clinical isolate, *C. jejuni* BH-01-014 [27]. Putative T6SS gene clusters were also identified in *C. coli* RM2228, *C. concisus* 13826, and *C. rectus* RM3267 (Figure 1). Twelve of 13 genes identified in the T6SS-positive *C. jejuni* strains exhibited high sequence similarity to genes that were considered core genes of T6SS, whereas a single gene, COG3456, was considered an accessory gene [3]. The list of these orthologs, with the corresponding Cluster of Orthologous Groups (COG) identification number and their potential functions, is presented in Table 1. Despite the presence of 13 T6SS genes, a gene encoding a core component, ClpV, which is present in the majority of previously characterized T6SS [3], was absent from the T6SS gene clusters of *C. jejuni* and *Campylobacter* spp. analyzed (Table 1).

**Table 1:** List of T6SS orthologs of *C. jejuni* ATCC 43431 and their proposed functions [3], [24].

T6SS orthologue ID	Gene name	Proposed functions	Size (amino acids)	NCBI protein sequence ID of *C. jejuni* ATCC 43431
**COG3501**	*vrgG*	Membrane penetrating component, effector	869	AAS98979
**COG3515**	*impA/vasJ*	Unknown function	356	AAS99033 (link to AAS99031 and AAS99032)
**COG3516**	*impB*	Form tubular structure resembling tail sheath of bacteriophages	141	Present (linked to AAS99033 and AAS99098)
**COG3517**	*impC*	Form tubular structure resembling tail sheath of bacteriophages	484	AAS99098 (link to AAS99096 and AAS99097)
**COG3518**	*vasS*	Bacteriophage baseplate GP-25 like lysozyme	135	AAS99097 (link to AAS99096 and AAS99098)
**COG3519**	*impG*	Unknown function	573	AAS99096 (link to AAS99097 and AAS99098)
**COG3520**	*impH/vasB*	Unknown function	302	Present (linked to AAS98979, AAS99033)
**COG3521**	*vasD*	Outer membrane lipoprotein involved in stabilizing T6SS	148	AAS99032 (link to AAS99031 and AAS99033)
**COG3522**	*impJ/vasE*	Unknown function	465	AAS99075 (AAS99031) (link to AAS99076)
**COG3523**	*icmF/impL*	Inner membrane protein involved in T6SS stabilization	1175	AAS98968, AAS98977
**COG3455**	*ompA/motB/dotU/impK/vasF*	Inner membrane protein involved in T6SS stabilization	222	AAS98993 (link to 98992)
**COG3456**	*fha1/impL/vasC*	Core scaffolding protein, phosphorylated by serine-threonine kinase	298	AAS98989
**COG3157**	*hcp1*	Membrane penetrating component, effector	171	AAS98992 (link to 98993)

The initial effort to sequence the genome of *C. jejuni* 43431 identified 11 conserved genes of T6SS [25]. However, COG3516 and COG3520 that are present in most of the *Campylobacter* spp. examined were not detected. To detect the two orthologs via polymerase chain reaction (PCR) amplification in *C. jejuni* 43431, a set of primers was designed based on COG3516 and COG3520 sequences of *C. coli* RM2228. The amplicons produced were of the expected size. Subsequently, COG3516 and COG3520 were verified by DNA sequencing, reconfirming their presence in *C. jejuni* 43431 (Table 1).

By *in silico* comparison of putative T6SS gene clusters among *Campylobacter* spp. as well as PCR and DNA sequencing, we determined that 12 core genes and the single accessory gene of *C. jejuni* 43431 are tightly linked and clustered in a discrete region of the genome (Figure 1). While the organization of certain core genes is highly conserved, the arrangement of others varies considerably among *Campylobacter* spp. (Figure 1). Interestingly, the arrangement of T6SS in *C. jejuni* 43431 is the exact replica of that in *C. coli* RM2228. However, genes within the T6SS cluster of *C. jejuni* 414 have undergone extensive rearrangement, resulting in noticeable differences in the T6SS cluster of this strain and *C. jejuni* 43431.

Within the T6SS clusters of *C. jejuni* and *C. coli* RM2228, five genes appear to form two distinct operons: *icmF* and its downstream gene, COG3456, as well as COG3517, COG3518, and COG3519. The coding sequences of the genes comprising each putative operon are arranged in the same orientation and their stop and start codons overlap with each other, indicating that these genes may utilize the same promoter and are co-transcribed. The rest of the genes within the cluster including *hcp* and *vrgG* appear to be stand-alone transcriptional units (Figure 1). In *C. jejuni* 43431 and *C. coli* RM2228, *hcp* is flanked on one side by the putative *icmF*/COG3456 operon and on the other side by COG3455, COG3521, and COG3522, all encoding the T6SS core components (Figure 1).

As previously mentioned, *hcp* and *icmF* are highly conserved and are essential components of T6SS [3]. Therefore, these genes were deleted in *C. jejuni* 43431 and the resulting mutants were characterized to elucidate the T6SS function (Figure S1). Based on the genetic arrangement of the T6SS gene cluster, deletions of *hcp* and *icmF* are unlikely to affect the function of downstream genes that are not involved in T6SS (Figure 1 and Figure S1C).

**Figure 2:**
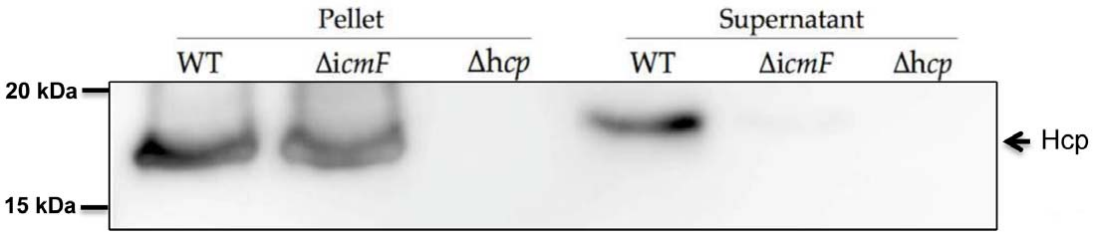
Western blot analysis of Hcp in WT *C. jejuni*, the Δ*hcp* mutant, and the Δ*icmF* mutant. Equal numbers of WT, Δ*hcp* and Δ*icmF* mutants were collected (see **Material and Methods**). Pellet and supernatant were separated and used in immunoblot assays with an anti-Hcp antibody. The estimated molecular weight of Hcp is 18 kD. The results are representative of three independent experiments.

A robust indicator of a functional T6SS in T6SS-harboring bacteria is the presence of the secreted substrate, Hcp, in the culture media [28]. To validate the secretion function of *C. jejuni* T6SS and characterize the functional defect of T6SS in Δ*hcp* and Δ*icmF* mutants, immunoblots for Hcp were performed. In the wild type (WT) *C. jejuni*, an expected 18 kD protein corresponding to *C. jejuni*'s Hcp was detected in both pellet and supernatant fractions (Figure 2). In the Δ*icmF* mutant, Hcp was present in both the pellet and the supernatant; however, the level of Hcp in the supernatant was substantially less than that in WT (Figure 2). In contrast, Hcp was completely absent from both pellet and the supernatant fractions of the Δ*hcp* mutant. This result indicates that the anti-Hcp antibody can detect *C. jejuni* Hcp with high specificity. Furthermore, IcmF is required for the secretion of Hcp, confirming the previously demonstrated role of IcmF in T6SS. These results strongly suggest that *C. jejuni* possesses a functional T6SS.

### *C. jejuni* T6SS mediates growth inhibition in response to high physiological concentrations of deoxycholic acid (DCA)

T6SS plays crucial roles in bacterial growth, motility and adaptation to environmental perturbations [11], [14], [15], [16]. Δ*hcp1* and Δ*icmF1* mutants of *C. jejuni* 43431 grew and swarmed at the rate comparable to that of WT (Figure S2). Furthermore, no significant difference in growth responses to NaCl, saponin and various classes of antibiotics, including tetracycline, gentamicin, fluoroquinolones, B-lactam (e.g., ampicillin, amoxicillin and cephalothin), sulfonamides and vancomycin, was observed between WT and the mutants (Table 2).

**Table 2:** Responses of WT *C. jejuni* ATCC 43431, Δ*hcp* and *ΔicmF* mutants, and the complemented Δ*hcp1* (*hcp1*(*hcp*+)) to bile salts, NaCl, saponin, different pH and antibiotics.

Agents/antibiotics	WT	*Δhcp1*(*hcp*+)	*Δhcp1*	*Δhcp2*	*ΔicmF1*	*ΔicmF2*
Cholic acid (MIC, µg/ml)	40,000	40,000	40,000	40,000	40,000	40,000
Deoxycholic acid (MIC, µg/ml)	12,000	15,000	>60,000	>60,000	>60,000	>60,000
Ox bile (MIC, µg/ml)	50,000	50,000	50,000	50,000	50,000	50,000
Ampicillin	S(29)	S(29)	S(29)	S(29)	S(29)	S(29)
Amoxycillin/clavulonic acid	S(38)	S(38)	S(38)	S(38)	S(38)	S(38)
Cephalothin	R(0)	R(0)	R(0)	R(0)	R(0)	R(0)
Gentamicin	S(29)	S(29)	S(29)	S(29)	S(29)	S(29)
Tetracycline	S(32)	S(32)	S(32)	S(32)	S(32)	S(32)
Enrofloxacin	S(38)	S(38)	S(38)	S(38)	S(38)	S(38)
Trimethoprim/sulfomethazole	S(24)	S(24)	S(24)	S(24)	S(24)	S(24)
Nalidixic acid	S(31)	S(31)	S(31)	S(31)	S(31)	S(31)
Vancomycin	R(0)	R(0)	R(0)	R(0)	R(0)	R(0)
NaCl (MIC, µg/ml)	10,000	10,000	10,000	10,000	10,000	10,000
Saponin (MIC, µg/ml)	15,000	15,000	15,000	15,000	15,000	15,000
pH (non-lethal range)	5–9	5–9	5–9	5–9	5–9	5–9

( ): approximate diameter of zone of growth inhibition in millimeter. Sensitivity (S) versus resistance (R) to antibiotics was determined based on the zone diameter value for *Enterobacteriaceae* reported by the manufacturer (BD, BBL). Experiments were performed on three independent occasions.

**Figure 3:**
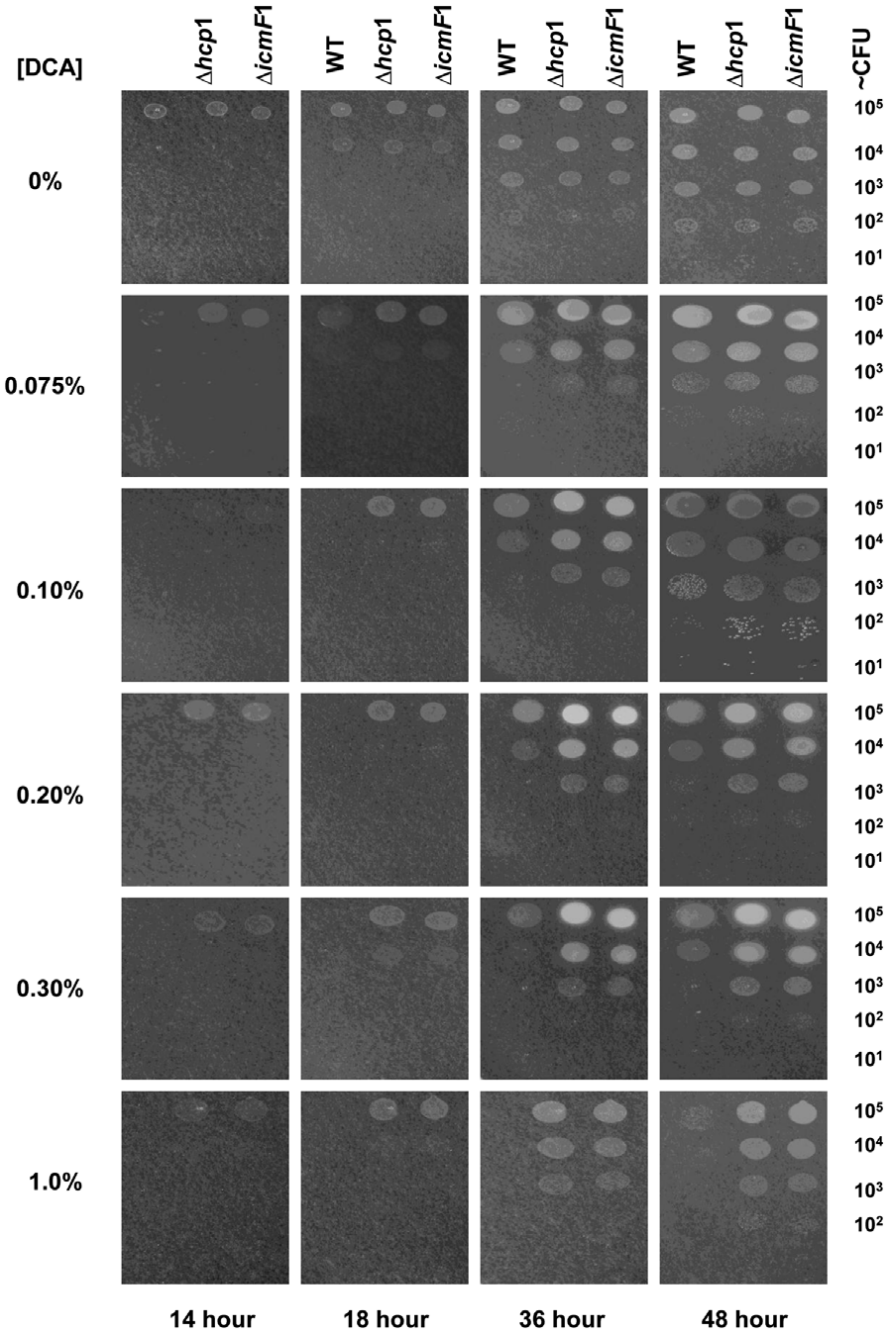
Growth dynamics of WT *C. jejuni*, the Δ*hcp1* mutant, and the Δ*icmF1* mutant in various sub-inhibitory concentrations of DCA. Ten-fold serial dilutions of WT and the mutants were spotted on agar plates lacking or supplemented with DCA at concentrations ranging from 0% to 1%. Growth was inspected at 14, 18, 36 and 48 hours after incubation under microaerobic conditions. The results are representative of three independent experiments.

As an enteric pathogen, *C. jejuni* must cope with the antimicrobial effects of bile salts secreted into the intestinal tract [29]. Growth responses of WT and the mutants in bile acids, specifically cholic acid, deoxycholic acid (DCA) and ox bile, were evaluated. Cholic acid, a primary bile acid generated by hepatocytes, and DCA, a bacterial metabolite of cholic acid, represent two major components of the bile salt found in the human intestine [30], [31]. Ox bile contains a mixture of conjugated and unconjugated bile acid derivatives, such as taurocholic acid, glycocholic acid, deoxycholic acid, and cholic acid [32], allowing simultaneous evaluation of responses to a variety of bile acid derivatives. A significant difference in growth responses to DCA was observed between WT and the mutants, while growth responses to cholic acid and ox bile were similar between WT and the mutants as shown by their similar minimum inhibitory concentrations (MICs) of these bile salts (Table 2). During a 48 hour-period, WT and the mutants displayed the same growth response at the range of DCA concentration of 0%–0.05% (Figure S3A), which corresponds to the lower range of physiological concentration of DCA found in the human large intestine [31], [33]. However, at the higher range of physiological concentration of DCA (0.075%–0.2%), found in the small intestine [33]–[35] and the concentrations above this physiological range (0.3%–1%), growth inhibition was observed in WT (Figure 3). In contrast, growth of the mutants was maintained at the level comparable to those grown without DCA at all concentrations tested throughout the 48 hours (Figure 3). As early as 14 hours, about a 10-fold reduction in growth can be detected in WT relative to the mutants at DCA concentrations of ≥0.075%. By 18 hours, growth of WT was found to be significantly reduced by <10-fold at 0.075% DCA and by 10- to 100-fold at 0.1% DCA relative to the mutants, while no visible sign of growth could be detected in WT at DCA concentrations of ≥0.2% (Figure 3). Interestingly, when *C. jejuni* growth was tabulated at 36 and 48 hours, growth of WT was observed in all concentrations tested (Figure 3). However, the growth level of WT was restored to levels comparable to those of the mutants at 0.75–0.2%, but not at concentrations of >0.2% (Figure 3). This difference in growth dynamics between WT and the mutants, in response to physiological concentrations of DCA, was also observed in liquid culturing conditions (Figure S3B).

**Figure 4:**
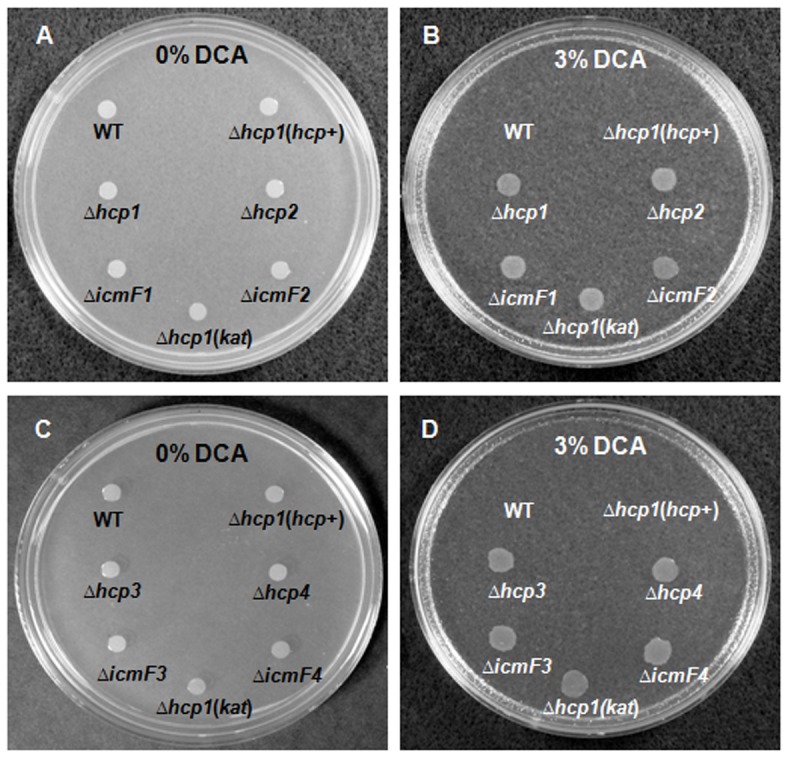
Growth responses of WT *C. jejuni*, Δ*hcp* and Δ*icmF* mutants, and the complemented Δ*hcp1* strain, Δ*hcp1*(*hcp+*), on agar free of or supplemented with 3% DCA. (A) WT, Δ*hcp* and Δ*icmF* mutants harboring *cat* (*hcp1*, *hcp2*, *icmF1*, *icmF2*), complemented Δ*hcp1* strain, Δ*hcp1*(*hcp+*), and the control Δ*hcp1* mutant harboring *kat* (Δ*hcp1*(*kat*)) grown on a DCA-free agar and (B) supplemented with 3% DCA. (C) WT, Δ*hcp* and Δ*icmF* mutants harboring *kat* (*hcp3*, *hcp4*, *icmF3*, *icmF4*), Δ*hcp1*(*hcp+*) and Δ*hcp1*(*kat*) grown on a DCA-free agar and (D) supplemented with 3% DCA.

To further assess the differences in DCA response, the MICs of DCA for WT and the mutants were determined. WT had an MIC of 1.2%. In contrast, Δ*hcp1* and Δ*icmF1* mutants had MICs of >6% (Table 2). We were unable to determine the exact MICs of the mutants due to solubility issues that were encountered when DCA concentrations reached 6%. To determine if the DCA-resistant phenotype observed in the mutants was the result of T6SS deficiency, we complemented the Δ*hcp1* mutant in *trans* by integrating the WT *hcp* under the control of a chloramphenicol acetyltransferase (*cat*) promoter into the 16S–23S rRNA spacer region of the *Δhcp1* mutant's chromosome (Figure S1A and S1D), thereby creating a complemented strain, Δ*hcp1*(*hcp*+), which over-expressed *hcp* at levels 3 times that of WT (Figure S4). Like the WT, the complemented strain failed to grow in media supplemented with 3% DCA, a concentration that exceeds the MIC of WT (Figure 4). However, the MIC of DCA for the complemented strain was slightly higher than that of WT (MICs: 1.5% in Δ*hcp1*(*hcp*+) versus 1.2% in WT, Table 2). The growth response to DCA of the control Δ*hcp1* mutants harboring kanamycin-resistance cassette (*kat*), which was also integrated into the 16S–23S spacer region, was similar to that of the mutants suggesting that integration of *kat* into the 16S–23S is not responsible for the DCA-sensitive phenotype observed in Δ*hcp1*(*hcp*+) (Figure 4B and 4D). These results indicate that a defective T6SS is responsible for the DCA-resistant phenotype observed in the mutants. Taken together, our data suggest that the presence of T6SS imposes a growth constraint at DCA concentrations of ≥0.075%, particularly during the initial exposure to DCA. Despite the initial growth inhibition, T6SS-harboring *C. jejuni* is capable of restoring its growth at the later stage, indicating that the bacteria are capable of adapting to the upper physiological range of DCA.

### Deficiency in T6SS transcriptionally down-regulates *C. jejuni* multidrug transporter gene during initial exposure to DCA

**Figure 5:**
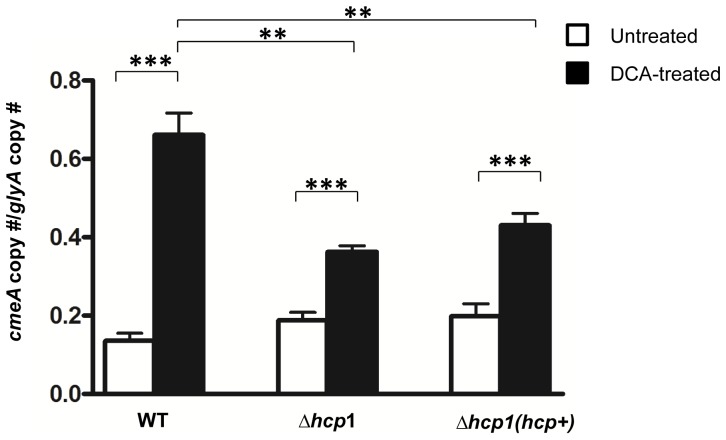
Expression levels of *cmeA* in WT *C. jejuni*, the Δ*hcp1* mutant, and the complemented Δ*hcp1* strain, Δ*hcp1*(*hcp+*), grown in liquid media free of or supplemented with 0.1% DCA after 14 hours of growth. The Y axis represents the ratio of *cmeA* copy number and the copy number of a house keeping gene, *glyA*, previously shown to not be affected by DCA [55]. The graph represents results from two independent experiments. Error bars represent the standard error of the mean. P value: **≤0.01, ***≤0.001.

Previous studies suggest that an active secretion system may enable unconjugated bile salts, such as DCA, to enter the bacterial cell through an opened secretion channel, subsequently inhibiting bacterial growth [36]. *CmeABC*, which encodes *C. jejuni* multidrug/bile salt efflux transporter, is positively regulated by DCA [37] and serves as an excellent molecular marker for monitoring changes in the intracellular DCA concentrations. We investigated whether *C. jejuni* T6SS mediates intracellular influx of DCA causing cell growth arrest by examining the expression level of *cmeA*, a component of *cmeABC*, in WT and the *Δhcp1* mutant grown in 0.1% DCA at 14 hours, a period when growth of the DCA-treated WT was severely inhibited, while the growth of the DCA-treated *Δhcp1*mutant was not (Figure 3). The transcript level of *cmeA* did not differ significantly between WT and the mutants grown in DCA-free media (Figure 5). In contrast, the transcript level of *cmeA* of the DCA-treated WT was 1.5 times that of the DCA-treated *Δhcp1* mutant (p≤0.01). Furthermore, the DCA-treated WT had a *cmeA* level that was 4.8 times that of the untreated WT (p≤0.001) (Figure 5). In contrast, the *cmeA* transcript level of the DCA-treated *Δhcp1* mutant was only 1.9 times (p≤0.001) that of the untreated-Δ*hcp1* mutant. The DCA-treated complemented strain, which had a DCA susceptibility comparable to that of WT, had a *cmeA* transcript level that was 2.2 times (p≤0.001) that of the untreated complemented strain (Figure 5). These results support our hypothesis that T6SS mediates intracellular influx of DCA to inhibit *C. jejuni* growth.

### *C. jejuni cmeA* and T6SS are sequentially modulated during adaptation to deoxycholic acid

**Figure 6:**
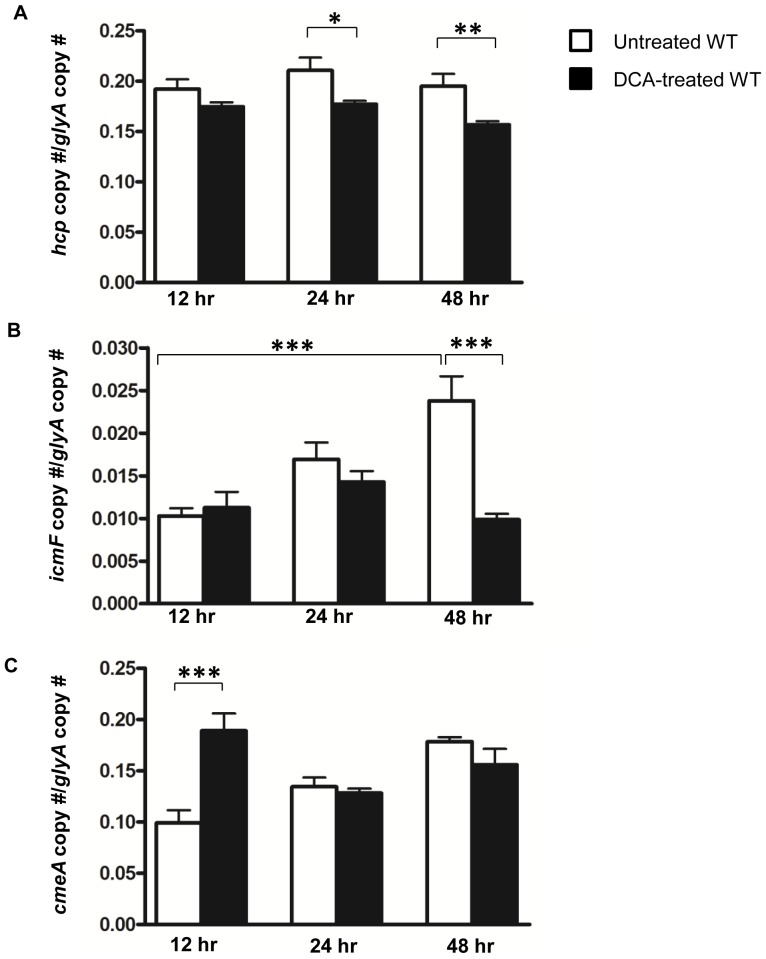
Temporal expression patterns of *hcp*, *icmF*, and *cmeA* during growth of *C. jejuni* in biphasic culture media free of or supplemented with 0.1% DCA. The transcript levels of (A) *hcp*, (B) *icmF*, and (C) *cmeA* of WT *C. jejuni* grown in the presence or absence of 0.1% DCA at 12, 24 and 48 hours of growth. The Y axis represents the ratio of *hcp*, *icmF* or *cmeA* copy number and the copy number of a house keeping gene, *glyA*. The graphs represent results from at least three independent experiments. Error bars represent the standard error of the mean. P value: *≤0.05, **≤0.01, ***≤0.001.

If the expression of T6SS inhibits *C. jejuni* growth by mediating DCA influx, but a defective T6SS enables growth by limiting DCA influx, down regulation of T6SS may serve as a mechanism for ensuring survival of T6SS-harboring *C. jejuni* in DCA. We next investigated the relationship between T6SS and the bile efflux transporter by characterizing temporal expression patterns of *hcp*, *icmF* and *cmeA* in WT during growth in biphasic culture media free of or supplemented with 0.1% DCA. We targeted three time points—12 hours when growth of the DCA-treated WT was inhibited; 24 hours; and 48 hours when growth was restored (Figure 3). In DCA-free media, *hcp* and *icmF* were transcribed throughout the entire growth phase. *hcp* was expressed at a steady level throughout the entire 48 hours, whereas transcript levels of *icmF* showed an increasing trend from 12 to 48 hours (Figure 6A). Compared to *hcp*, the transcript level of *icmF* was significantly lower at all time-points (Figure 6A and 6B). No significant difference in *hcp* and *icmF* transcript levels was observed between the DCA-treated WT and the untreated controls at 12 hours (Figure 6A and 6B). However, the *hcp* transcript level of the DCA-treated WT was significantly less than that of the untreated control at 24 (p≤0.05) and 48 hours (p≤0.01, Figure 6A). Likewise, the *icmF* transcript level of the DCA-treated WT was significantly decreased compared to that of the untreated control at 48 hours (p≤0.001, Figure 6B). In contrast, the DCA-treated WT had a *cmeA* transcript level that was 2 times that of the untreated WT at 12 hours (p≤0.001) (Figure 6C). However, no significant difference in the transcript levels of *cmeA* was detected between the DCA-treated and the untreated WT at 24 and 48 hours (Figure 6C). The above results demonstrate that T6SS is down-regulated when *C. jejuni* growth is restored, supporting the view that down-regulation of T6SS function enables *C. jejuni* to survive in DCA. The observed initial up-regulation of *cmeA* further substantiates the notion that T6SS mediates DCA influx. Considering the roles of *cmeA* in bile resistance, these results indicate that the coordinated transcriptional regulation of the bile efflux transporter and T6SS may represent, in part, an adaptive mechanism for ensuring *C. jejuni* survival in high physiological concentrations of DCA.

### *C. jejuni* T6SS is required for adherence to and invasion of *in vitro* T84 human colonic epithelial and murine RAW 264.7 macrophage cell lines

**Figure 7:**
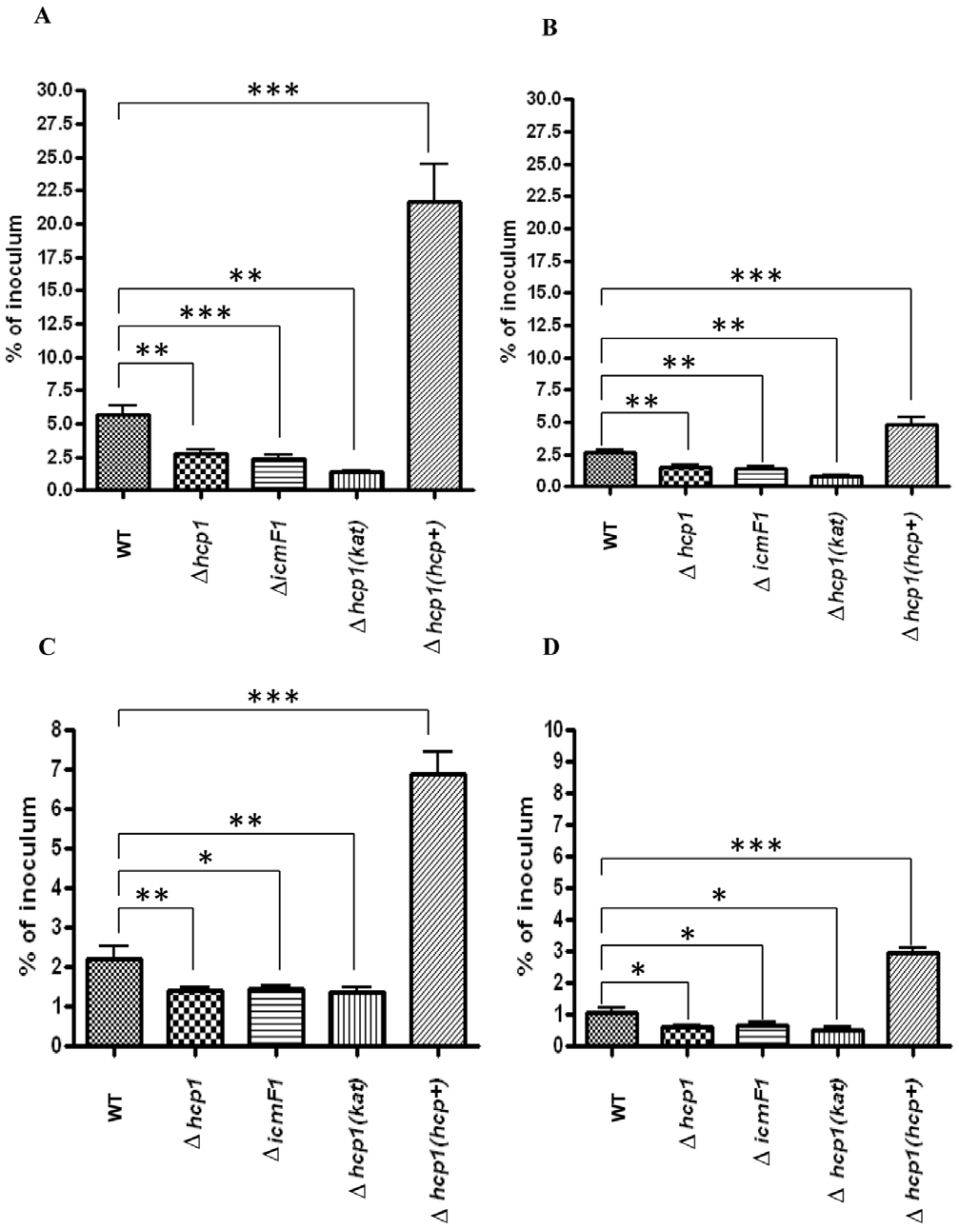
Adhesion and invasion efficacy of WT *C. jejuni*, the Δ*hcp1* mutant, the Δ*icmF1* mutant, and the complemented Δ*hcp1* strain, Δ*hcp1*(*hcp+*), in human T84 colonic epithelial and RAW 267.4 macrophage cell lines. (A) T84 adhesion efficacy, B) T84 invasion efficacy, (C) RAW 267.4 macrophage adhesion efficacy and (D) RAW 267.4 macrophage invasion efficacy. The graph represents results from three independent experiments. Error bars represent the standard error of the mean. P value: *≤0.05, **≤0.01, ***≤0.001.

Recent studies in *E. coli* suggest that T6SS promotes host cell adhesion and invasion, the two fundamental processes required for host colonization and the attainment of full virulence [23], [38]. To explore the roles of *C. jejuni*'s T6SS in these processes, we evaluated the cell adhesion and internalization potentials of Δ*hcp1* and Δ*icmF1* mutants and WT in *in vitro* T84 colonic epithelial and RAW 264.7 macrophage cell lines. Compared to the parental WT, the mutants showed a significant reduction, by approximately 50% of that observed in WT, in their abilities to adhere to and invade the T84 cell line (Figure 7A and 7B). Likewise, the RAW 264.7 adherence and invasion efficacy/number of bacteria phagocytized by RAW 264.7 of the mutants was approximately 50% lower than that of WT (Figure 7C and 7D). Remarkably, the complemented strain, which over-expressed *hcp*, adhered to and invaded the T84 and RAW 264.7 at levels of approximately 4 times and 2 to 3 times that of WT, respectively (Figure 7A and 7B). The control Δ*hcp1* mutants harboring *kat* exhibited cell adhesion and invasion characteristics similar to the mutants (Figure 7). The higher rates of cell adhesion and invasion exhibited by the complemented strain were likely due to the overexpression of *hcp*, but not the higher rate of cell proliferation and motility as the complemented strain grew and swarmed at the rate comparable to WT and the mutants (Figure S2). These results indicate that the reduced efficacy of cell adhesion and invasion observed in the Δ*hcp* mutant resulted from *hcp* deficiency. Collectively, these data suggest that *C. jejuni* T6SS promotes host cell adhesion and invasion and that *hcp*-mediated cell adhesion and invasion operate in a dose-dependent fashion.

### *C. jejuni* T6SS is required for persistent colonization in IL-10-deficient mice

**Figure 8:**
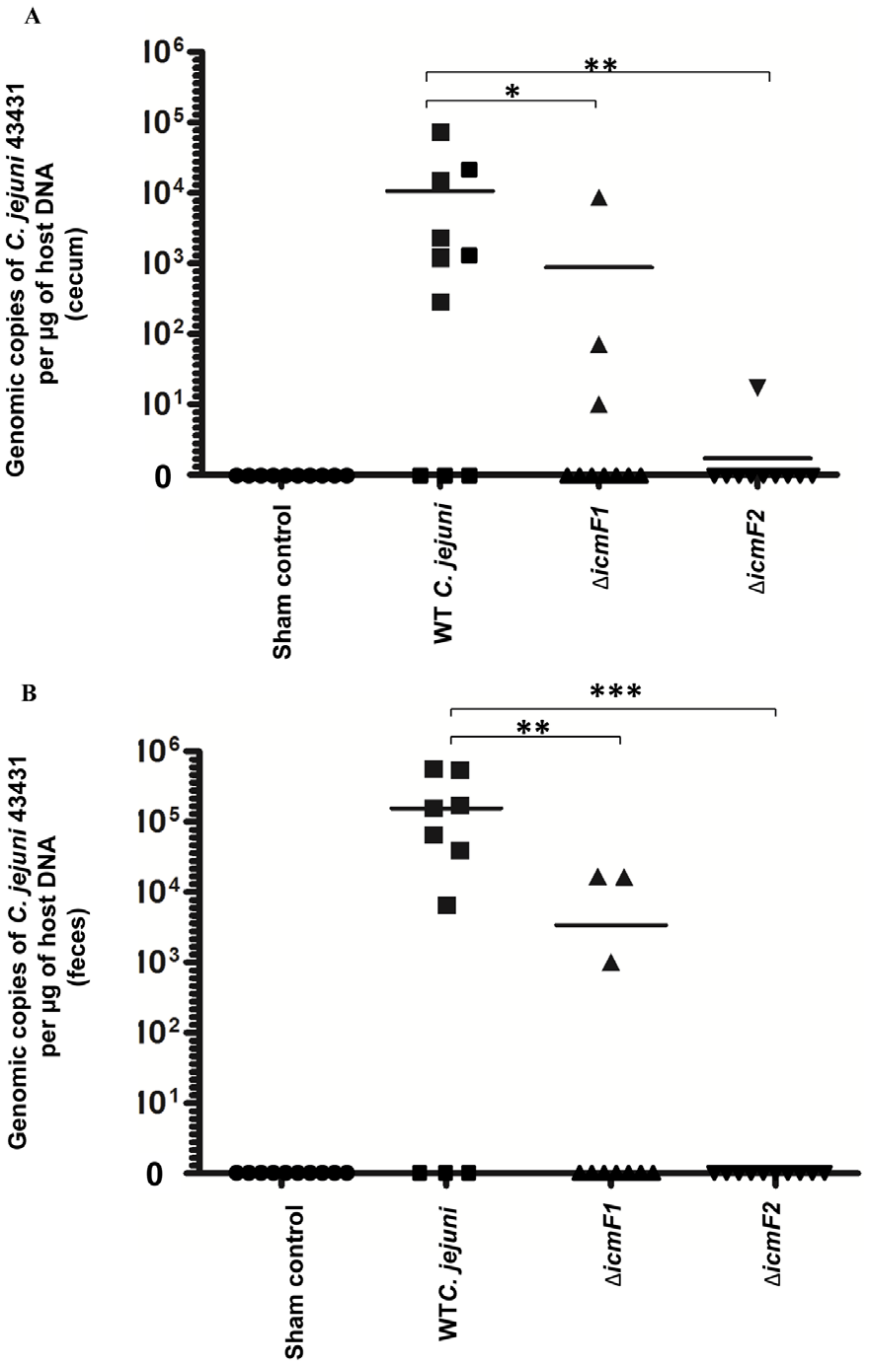
Colonization potential of WT *C. jejuni*, the Δ*icmF1* mutant, and the Δ*icmF2* mutant in IL-10-deficient mice at 30 days post-infection. The number of WT *C. jejuni* 43431 and the mutants in the cecum (A) and the feces (B) of individual mice were quantified by Q-PCR (see **Material and Methods**) and the values plotted. A solid line represents the mean value for each group. P value: *≤0.05, **≤0.01, ***≤0.001.

Although the above findings suggest that T6SS defects may not affect *C. jejuni*'s ability to multiply and survive when exposed to DCA in the small intestine, the defects are likely to prevent C. *jejuni* from adhering to and invading the epithelial lining of the colon. As host cell adhesion and invasion are crucial for enteric pathogens to colonize and exert their pathogenic potential and/or establish persistence, we hypothesized that T6SS defects would adversely affect *C. jejuni in vivo* colonization potential. We compared the colonization potential of WT and T6SS-deficient strains in the B6.129P2-*IL-10^tm1Cgn^* mice, proven to be a useful model for evaluating *C. jejuni* colonization of the large intestine [39], [40]. *C. jejuni* 43431 and 2 independent *C. jejuni* Δ*icmF* mutants (Δ*icmF1* and Δ*icmF2*) were used to experimentally inoculate 6 to 7 week-old male and female IL-10-deficient mice (see **Material and Methods**). At 30 days post inoculation (p.i.), mice were euthanized and their cecal tissues and fecal samples were quantitatively analyzed for *C. jejuni* by q-PCR. *C. jejuni* was not detected in either the cecal tissues or the feces of 10/10 sham-dosed control mice (Figure 8A and 8B); however, WT *C. jejuni* was detected in the cecal tissues of 7/10 mice at the mean copy number of C. *jejuni*/µg of host DNA of 10669±7120 (mean±standard error). In contrast, the *C. jejuni* Δ*icmF1* mutant was detected in only 3/10 mice at 877±867copy numbers/µg and in 1/10 mice inoculated with the *C. jejuni* Δ*icmF2* mutant at 1.7±1.7 copy numbers/µg (Figure 8A). This was consistent with the results obtained from the fecal samples (Figure 8B): WT: 156522±70250 copy numbers/µg (7/10 mice) versus Δ*icmF1*: 3405±2189 copy numbers/µg (3/10 mice) and Δ*icmF2*: 0 copy number/µg (10/10 mice). The finding that numbers of *C. jejuni* were significantly reduced in mice infected with *ΔicmF* mutants compared to those infected with WT indicates that T6SS is instrumental in *C. jejuni* to establish a persistent colonization.

## Discussion

In our studies, we demonstrated that the human clinical isolate, *C. jejuni* ATCC 43431, possesses a T6SS gene cluster that encodes a functional secretion system. To our knowledge, this is the first report of a functional characterization of T6SS in *Campylobacter jejuni*. Importantly, our study reveals the role of T6SS in host cell adhesion, invasion, and a novel role of T6SS in adaptation to a specific bile salt, deoxycholic acid, which together contribute to the ability of this strain of *C. jejuni* to establish *in vivo* colonization.

Unlike other known T6SSs, the T6SS of *C. jejuni* and *Campylobacter* spp. appears to lack ClpV belonging to AAA+ family of ATPases, which was hypothesized to provide the energy toT6SS and is present in most T6SS-harboring bacteria [41]. This lack of ClpV in the T6SS of *C. jejuni* is shared with *Francisella tularensis*, *H. hepaticus*, *S. cholerasuis*, and *Burkholderia* spp. [8], [42]. Further analyses are required to determine how the lack of ClpV is compensated for in *C. jejuni* and the aforementioned species. The variation in the arrangement of T6SS genes within T6SS clusters of different *Campylobacter* spp. may contribute to adaptation to specific hosts or niches. Intriguingly, the T6SS of *C. jejuni* 43431 is the exact replica of *C. coli* RM2228, but is distinct from those of *C. jejuni* 414 and *C. jejuni* BH-01-0142. Furthermore, 3 of the 14 (21%) strains of *C. jejuni* analyzed in this study harbor T6SS gene clusters, while the rest of the *C. jejuni* strains, including the well characterized human clinical isolates, *C. jejuni* 81–176, *C. jejuni* 81116 and *C. jejuni* 11168, do not contain T6SS. Interestingly, both T6SS-positive strains, *C. jejuni* 43431 and *C. jejuni* BH-01-0142, belong to the same serodeterminant capsule of the HS3 serogroup, whereas the T6SS-negative strains, such as *C. jejuni* 81–176, belong to a distinct serogroup, HS23,36 [27]. Additional studies are required to determine whether T6SS is conserved among strains within the HS3 serogroup and how T6SS may contribute to their pathogenesis.

The *in vivo* survivability of enteric pathogens depends on their ability to colonize and interact with their target host [42]. Our results suggest that *C. jejuni* T6SS is involved in these processes with Hcp acting as a key mediator. Hcp-mediated cell adhesion and invasion appears to operate in a dose-dependent fashion. Over-expression of *hcp* in the complemented strain further enhanced cell adhesion and invasion compared to WT and, to a greater extent, the mutants. In the meningitis-causing *E. coli* K1 strain RS218, the secreted Hcp1 and the non-secreted Hcp2 encoded by its T6SS gene cluster promote invasion and adhesion, respectively, to human brain microvascular endothelial cells (HBMEC) [23]. Since it was not secreted, it was proposed that Hcp2 acts as a structural protein facilitating HBMEC adhesion by interacting with the host cell surface protein. In contrast, the secreted Hcp1 serves as an effector protein, which upon entering the host intracellular compartment, induces actin cytoskeletal rearrangement, cytokine production, and apoptosis. Unlike *E. coli*, *C. jejuni* 43431 contains only a single secreted Hcp that promotes both host cell adhesion and invasion. *C. jejuni* Hcp may, therefore, serve as both a structural protein that facilitates host cell adhesion and as an effector that promotes host cell invasion.

T6SS has been implicated in bacterial growth, motility, and survival under different stress conditions. Although *C. jejuni* T6SS components are constitutively expressed during growth under normal *in vitro* growth conditions, the T6SS is not essential for viability, growth, or motility. However, a functional T6SS increases susceptibility of *C. jejuni* to a bile salt, DCA. Alterations in the outer membrane permeability barrier can lead to increased permeability to DCA [43]. However, the WT's and the mutants' growth responses to hydrophobic (e.g. trimethoprim) and hydrophilic (e.g. ampicillin and tetracycline) antibiotics were similar, implying that T6SS has no impact on the integrity of the membrane permeability barrier. Thus, this mechanism is unlikely to contribute to the DCA-sensitive phenotype. The possible mechanism of T6SS-mediated DCA sensitivity is an increase of intracellular influx of DCA. This is based on the finding that *cmeA*, a bile efflux transporter gene previously shown to be positively regulated by DCA, was significantly up-regulated in the DCA-treated WT relative to both the untreated control and the T6SS-deficient strains during the initial exposure to DCA when *C. jejuni* growth was inhibited. The type IV secretion system (T4SS) of *E. coli* was implicated in mediating bile acid sensitivity, specifically to unconjugated bile acids, such as DCA and cholic acid, as well as sodium dodecyl sulfate [36]. It was proposed that T4SS and possibly other types of secretion systems, when transiently activated, allow macromolecules of a suitable size and chemistry, such as unconjugated bile acids, to passively enter the cells and inhibit cell growth. It remains to be determined whether a similar mechanism for *C. jejuni* T6SS exists.

Despite the severe growth inhibition at the extreme DCA concentrations, *C. jejuni* expressing T6SS is able to adapt to the inhibitory effect of physiological concentrations of DCA. Our results suggest that this is in part due to the sequential modulation of the bile efflux transporter and the T6SS activity, which is characterized by the initial up-regulation of *cmeA* and the subsequent down-regulation of *hcp* and *icmF*. It is likely that the initial increase in the bile efflux transporter activity enables *C. jejuni* to counteract the DCA influx mediated by T6SS, while the subsequent down-regulation of T6SS activity allows *C. jejuni* to survive by limiting DCA influx. Thus, the coordinated regulation of the bile efflux transporter and the T6SS may represent an adaptive mechanism for ensuring *C. jejuni* survival during exposure to the upper range of physiological concentrations of DCA.

Our *in vitro* analyses suggest that Hcp plays a pivotal role in promoting host cell adhesion and invasion since the over-expression of *hcp* enhanced cell adherence and invasion in the complemented *Δhcp1* strain. However, the complemented strain displayed a greater resistance to DCA as well as a lower *cmeA* level during the initial exposure to DCA compared to WT. This is most likely the result of altered stoichiometry caused by the constitutive and/or over-expression of a secreted component of T6SS in *trans* [44].

The human intestinal tract is characterized by a gradient of DCA concentration with the highest concentration found in the duodenum and the lowest in the distal colon. The intra-duodenal concentration of total bile salt can range from 6.9 (∼0.3%)–9.3 (∼0.4%) mM [35] and is gradually decreased to 2 mM (∼0.1%) in the lower ileum [34]. Relative to the small intestine, the total bile salt concentration is low in the cecum and was reported to be in the range of 0.14 mM (0.006%)–0.93 (0.04%) mM [31]. Of the total bile acid, the concentration of DCA can vary from 0% to more than 50% depending on the individual [33]. Based on the current understanding of this DCA concentration gradient, we proposed a model explaining how temporal and spatial regulation of T6SS in response to the gradient of DCA dictates T6SS-harboring *C. jejuni*'s colonization dynamics and pathogenic potential in the human gastrointestinal tract. After *C. jejuni* survives the acidic environment of the stomach and enters the small intestine, T6SS, which is constitutively expressed in the absence of DCA, allows DCA to enter *C. jejuni* resulting in cell growth arrest. The increase in the intracellular concentration of DCA first up-regulates *cmeA* and then down-regulates T6SS expression. Ultimately, these two convergent processes synergize to promote a reduction of intracellular DCA, thereby restoring homeostasis and *C. jejuni* growth. Although *C. jejuni* is able to grow and multiply in the small intestine, the relatively low level of *hcp* and *icmF* attenuates the ability of *C. jejuni* to adhere to and invade the small intestine. Thus, the T6SS-harboring *C. jejuni* would not be able to effectively colonize this region of the intestine. However, as *C. jejuni* reaches the proximal colon, where the concentration of DCA is non-inhibitory (<0.05%), the restriction on T6SS expression is released, enhancing the capability of *C. jejuni* to adhere to and invade the mucosal epithelia and macrophages. Consequently, *C. jejuni* containing T6SS can establish colonization and induce colonic inflammation.

Our proposed model of colonization dynamics is consistent with the clinicopathologic features reported in humans in which pancolitis was the most common and consistent pathologic characteristic [45]–[47]. Interestingly, some studies reported two distinct patterns of pathology – lesions confined to the large intestine and those distributed in both the small and the large intestine [48]. Given the great genetic variability among *C. jejuni* strains, it is reasonable to assume that certain strains of *C. jejuni* have evolved to colonize and invade both the small and large intestine, while other strains, such as T6SS-harboring strains, have evolved to effectively colonize the colon. Indeed, mice inoculated with *C. jejuni* 43431 showed higher levels of persistent colonization in the large intestine compared to those inoculated with the T6SS-defective mutants.

In summary, our *in vitro* analyses revealed that T6SS isogenic mutants displayed approximately a 50% reduction in cell adhesion and invasion compared to WT *C. jejuni*. There results were complemented by the *in vivo* studies demonstrating the importance of T6SS in establishing persistence colonization. Further, this study demonstrates the role of T6SS in mediating DCA sensitivity and suggests the functional plasticity of the T6SS –the regulation of bile acid adaptation and host colonization – providing novel insights into the roles of T6SS in *C. jejuni* pathogenicity.

## Materials and Methods

### Bacterial strains and culturing methods

*Campylobacter jejuni* subsp. *jejuni* serotype O:3 (ATCC®43431™, Manassas, VA) was propagated on blood agar containing 5% defibrinated sheep blood (Remel, Lexington, KS) or in biphasic growth media [49] containing an equal volume of Brucella agar and Brucella broth (BD, Franklin Lakes, NJ). When appropriate, the media were supplemented with chloramphenicol (Cm, 25 µg ml^−1^) and/or kanamycin (Kn, 50 ug ml^−1^). Bacterial culture were incubated in GasPak jar filled with microaerobic gas mixtures (10% H_2_, 10% CO_2_, 80% N_2_) in a humidified 37°C incubator as previously described [50].

### Molecular techniques

Bacterial genomic and plasmid DNA were isolated using a Roche High Pure PCR Template Preparation kit (Roche Applied Science, Indianapolis, IN) and QIAprep Spin Miniprep Kit (Qiagen, Valencia, CA), respectively, according to the manufacturer's recommendations. The sequences of T6SS orthologs of *C. jejuni* ATCC 43431 were obtained by PCR with primers listed in Table 3. PCR amplification using Expand High Fidelity PCR system (Roche Applied Science) was performed with Gene Amp PCR system 9700 (AB Applied Biosystems, Carlsbad, CA). DNA sequencing was accomplished using the ABI prism Genetic Analyzer 3500 (Invitrogen, Carlsbad, CA). *E. coli* strains Top10 was used as a recipient for cloning, mutagenesis, and plasmid propagation. *E. coli* was cultured in Luria-Bertani (LB) agar or broth (BD) with continuous shaking (250 rpm) at 37°C under aerobic conditions. When appropriate, the plates were supplemented with ampicillin (50 µg ml^−1^), Cm and/or Kn as mentioned above.

**Table 3:** Primers used in this study.

Primer name	Primer sequences
**Primers for confirmation of T6SS genes**	
*icmF1*	5′-TATAGGATCCGTGTTCTTCTAAATTTGAAAATGT-3′
*icmF2*	5′-TATACAGCTGTTATCGTACCTCTCCTTGGCGAT-3′
*icmF3*	5′-TATAGGATCCGAATTTAGAAAATTTTAATGTTGGCA-3′
*icmF4*	5′-TATACAGCTGTTAAAATTTTCTAAAATTTACTGGAA-3′
*hcp1*	5′-TATAGGATCCGACTCCTACTGGAGATTTGAAAGATT-3′
*hcp2*	5′-TATACAGCTGTTATTCTAAAGGGGTAGCAGCTGTT-3′
*COG3515-F*	5′-AAGGGAGAATGACTCATAGCCGCA-3′
*COG3516-F*	5′-ATGTCAGATGGATCTTATGCTC-3′
*COG3516-R*	5′-TTAATTTCCTTGCTCTTCTTG-3′
*COG3517-R*	5′-GCTCAAGTTGCACCACAACTTCCA-3
*COG3456-R*	5′-TCAATGCTTTTTGCATCCAC-3′
*COG3501-F*	5′-TCCTTACCTTAACCCTTCCATGCTC-3′
*COG3520-F*	5′CATCGTGTTTGGTTCCATTGGGCA-3′
*COG3520-R*	5′-ATGAATAATCTTACTTCATATAGT-3′
**Primers for Q-PCR**	
*hcp*RTPCR-*F*	5′-TGCCAAATGCGCAAGAGTCAAGT-3′
*hcp*RTPCR-*R*	5′-TTCCGCTAGTTCCAGCAGCAGTAT-3′
*icmF*RTPCR*-F*	5′-TCTGACATCGAATACCCACTTAGTGA-3′
*icmF*RTPCR*-R*	5′-GTATTTCATCGCTACTTGTAGGCTTA-3′
*cdtA*RTPCR*-F*	5′-AATGCATACAAGCACCTATTACAA-3′
*cdtA*RTPCR*-R*	5′-TATACTGCAGTTATCGTACCTCTCCTTGGCGATATA-3′
**Primers for generation of Δ** ***hcp*** ** and Δ** ***icmF*** ** mutants**	
*hcp5′BAMHI-F*	5′-TATAGGATCCTACTGAAGCGAGTATTGGCAA-3′
*hcp3′HINCII-R*	5′-TATAGTCGACTTGTTGCGGTTCTAAACCA-3′
*hcp5′HINCII-F*	5′-TATAGTCGACTTATAATTTATCTTAAATAATCCT-3′
*hcp3′XHOI-R*	5′-TATACTCGAGTATTAAGCTATGCAAT-3′
*icmF5′BAMHI-F*	5′-TATAGGATCCATCAAGATTATTTATAACTGCTT-3′
*icmF3′HINCII-R*	5′-TATAGTCGACTGTAAATGAACTCATCAGCTT-3′
*icmF5′HINCII-F*	5′-TATAGTCGACATCACCAATGCAGGCAATCT-3′
*icmF3′XHOI-R*	5′-TATACTCGAGTTATTTAATTCACTAGCACCGTT-3′
**Primers for generation of the complemented Δ** ***hcp1*** ** strain**	
*16S-23SBAMHI-F*	5′-TATAGGATCCACGCAATACCGCGAGGT-3′
*16S-23SNOTI-R*	5′-TATAGCGGCCGCTGCATAATGCTATATGCTACT-3′
*pCATBAMHI-F*	5′-TATAGGTACCTCTAGAGTGATATAGATTGAAAAGTGG-3′
*pCATECORI-R*	5′-TATAGAATTCGTCGACGTAAATTCCGATTTGTTGAT-3′
*cjhcpHINCII-F*	5′TATAGTCGACTTAAGAAGGAGATATACATATGGCTGAACCAGCGTT-3′
*cjhcpXHOI-R*	5′-TATACTCGAGTTAAGCTTTGCCCTCTCTCCA-3′
*katECORIXHOI-F*	5′TATAGAATTCTCGAGAAGGAGATATACATATGATTGAACAAGATGGAT-3′
*KatXBAI-R*	5′-TATATCTAGATCAGAAGAACTCGTCAAGAA-3′

### Anti-Hcp antibody production

Custom anti-Hcp antibody was produced against the most antigenic epitope (CSGQPSGQRVHKPFSF) that is highly conserved among T6SS-positive *Helicobacter* spp. and *Campylobacter* spp. by Covance Inc. (Denver, PA) from the initial stage of Hcp peptide production to affinity purification of the antibody. Two New Zealand white rabbits were used in the standard 77-day protocol that was approved by the MIT Animal Care Committee. Bleeds were checked against purified Hcp peptide by ELISA and Western blot analysis. Antibody was enriched by affinity purification and was used for the subsequent analyses.

### Hcp secretion assay and Western Blot analysis

The Hcp secretion assay was performed according to a previously published protocol [10]. Briefly, equal numbers of WT *C. jejuni* 43431 and Δ*hcp1* and Δ*icmF1* mutant cells were harvested from liquid cultures at the logarithmic growth phase based on OD_600 nm_ readings and the supernatant was separated from the cells by centrifugation and passed through a 0.2 µm syringe filter (Millipore, Billerica, MA). Hcp was detected by incubating with the custom anti-Hcp primary antibody (Covance, as described previously, 1∶1000 dilution) for 1 hour at room temperature, followed by incubation with an HRP-conjugated goat anti-rabbit IgG secondary antibody (1∶10,000 dilution; Zymed, CA) for 1 hour at room temperature. Protein was transferred to a 0.2 µm PVDF membrane (Biorad, Hercules, CA) with a Trans-Blot Turbo transfer system (Biorad). Antibody binding was visualized with a Lumigen PS-3 chemiluminescent HRP detection kit (Lumigen, Southfield, MI) according to the manufacturer's recommendations.

### Generation of Δ*hcp* and Δ*icmF* mutants

Four Δ*hcp* mutants (2 mutants with *cat* oriented in opposite orientation, Δ*hcp1* and Δ*hcp2*, and 2 mutants with *kat* oriented in opposite orientation, Δ*hcp3* and Δ*hcp4*) and 4 Δ*icmF* mutants (*ΔicmF1*, *ΔicmF2*, *ΔicmF3*, and *ΔicmF4*) were generated by allelic replacement via homologous recombination (Figure S1). To create plasmid constructs used to generate Δ*hcp* mutants, the 247-bp DNA fragment in the upstream 5′ region of the gene was amplified by PCR using the forward primer (*hcp5′BAMHIF*) and reverse primer (*hcp3′HINCIIR*). The 353-bp region located at the distal portion of 3′ region was amplified using the forward primer (*hcp5′HINCIIF*) and reverse primer (*hcp3′XHOIR*) (Table 3). The two PCR products were ligated into the BamHI and XhoI sites of the suicide vector pBluescript-II SK and the resulting plasmid was circularized at the HincII sites of the two PCR products. *cat* (750 bp) of *C. coli* and *kat* (1300 bp) were excised from pBluescript-II SK vector with HincII and ligated into the HincII sites of the constructs effectively deleting a large middle portion of the *hcp* gene. Restriction endonucleases were obtained from New England Biolab (Ipswich, MA). The ligation reactions were performed using T4 DNA ligase (New England Biolab) according to the manufacturer's recommendations. The constructs used to create the *ΔicmF* mutants were generated in similar fashion as described for Δ*hcp* mutants with *icmF*-specific primers (Table 3). The orientation of *cat* and *kat* was determined by restriction enzyme analyses and was subsequently confirmed by DNA sequencing.

Bacterial transformation was performed by electroporation as previously described with minor modifications [51]. All mutants were passaged five times to ensure the elimination of any WT contaminants. Authenticity of the mutants was verified by PCR (Figure S1A and S1B) using gene-specific primers listed in Table 3.

### Complementation of Δ*hcp1* mutants

The **Δ** *hcp1* mutant was complemented in *trans* by stably integrating WT *hcp* coding sequence under the control of the *cat* promoter into the 16S–23S rRNA intergenic spacer region of the Δ*hcp1* mutant chromosome by the method previously described [52]. The complementation construct was generated by PCR amplification of 5′ and 3′ regions of 16S–23S using primers listed in Table 3. The 16S–23S sequences were cloned into the suicide vector mentioned above. A *cat* promoter sequence was amplified from the aforementioned vector and cloned into the middle of the 16S–23S sequences. *hcp* and *kat* coding sequences engineered to contain ribosome binding sites were amplified with primers listed in Table 3 and cloned into the vector immediately downstream of the *cat* promoter effectively generating a *hcp*/*kat* operon. The construct was transformed into *E. coli* Top10 and positive clones were selected based on resistance to Kn. The construct was sequenced to ensure sequence correctness and was transformed into the Δ*hcp1* mutant by electroporation as previously mentioned. The complemented Δ*hcp1* strain (Δ*hcp1*(*hcp+*)) was selected based on the resistance to Kn and Cm and confirmed by PCR and DNA sequencing (Figure S1A).

### *In silico* analysis

We analyzed the proteome sets of the following, including *C. jejuni* strains and clinically relevant *Campylobacter* spp.: *C. jejuni*-81-176, *C. jejuni*-CG8421, *C. jejuni*-BH-01-0142, *C. Coli*-RM2228, *C. concisus* 13826, *C. rectus*, *C. jejuni*-S3, *C. jejuni*-RM1221, *C. jejuni*-M1, *C. jejuni*-ICDCCJ07001, *C. jejuni*-IA3902, *C. jejuni* Doylei-269, *C. jejuni*-1336, *C. jejuni*-81116, *C. jejuni*-43431, *C. jejuni*-NCTC-11168 and *C. jejuni* 414. For each, the respective available proteome set was downloaded from the EBI or the NCBI. One proteome set, i.e., that of *C jejuni* 43431, had to be retrieved from the genome sequencing contigs from NCBI, as there was no full proteome set available for downloading from either EBI or NCBI.

The proteome sets were then BLASTed against NCBI's Reference Sequence Project Protein database using blastp with default parameters. BLAST results were saved as XML files. Each BLAST XML file for the respective proteome set was used to filter for proteins homologous to known T6SS-related proteins (e.g., *icmF*, *hcp*, *vrg*, *imp*, *vas*) using a Perl script we wrote, which utilizes BioPerl's Bio::SearchIO module.

### Growth and motility assay

For the motility assay, overnight *C. jejuni* cultures were harvested, suspended in 1× PBS and diluted to OD_600 nm_ of 0.1. Two µl of the sample (∼10^5^ CFUs) was spotted in the center of semisolid motility agar plates containing Brucella broth and 0.3% (w/v) agar adjusted to pH of 7. The plates were cultured for 24 hours and inspected for halos formed on semisolid agar as an indicator of motility. For growth assays, overnight *C. jejuni* cultures were added to the biphasic growth media to achieve OD_600 nm_ of 0.1. The cultures were incubated under microaerobic conditions with continuous shaking (100 rpm) and OD_600 nm_ was measured at various time points. Three independent experiments were performed for growth and motility assays. Gram's staining was conducted to determine the purity of each sample. PCR with *hcp*- and *icmF*-specific primers were used to confirm the identity of WT, the mutants, and the complemented strains at the beginning and the end of the growth assays.

### Bile acids, salt (osmolarity), saponin and pH assays

Two µl (∼10^5^ CFUs) of overnight culture of WT and the mutants was spotted and grown on Brucella agar plates containing 1.5% agar adjusted to a pH ranging from 4–10 or on Brucella agar plates adjusted to pH of 7 supplemented with cholic acid (0%–5% (50 mg ml^−1^) w/v); Sigma Aldrich, St. Louis, MO), ox bile (0%–5%; Sigma), sodium deoxycholate (0%–6%; DCA; Sigma), NaCl (0%–5%; Sigma) and saponin (0%–3%; Sigma). The desired pH of the solution was achieved with 1 M HCl and 1 M NaOH. MICs of each factor tested were determined by identifying concentrations that completely inhibited *C. jejuni* growth after 48 hours of incubation. To evaluate the growth dynamic of WT and the mutants in the presence and the absence of DCA, fresh bacterial samples were adjusted to OD_600 nm_ of 0.1 and were subjected to 10-fold serial dilutions. Two µl dilution of each dilution was spotted on Brucella agar plate supplemented or not supplemented with 0.1%DCA and incubated for specified times before recording observations. To evaluate the growth dynamics of WT and the mutants in different DCA concentrations under liquid culturing condition, overnight bacterial cultures were seeded in 5 ml Brucella broth to achieve an OD_600 nm_ of 0.1 and incubated under microaerobic conditions until specified time points. The number of viable bacteria that were present in each sample and time point was determined by 10-fold serial dilutions, plating, and enumeration of CFUs. All experiments were conducted on three independent occasions.

### Antimicrobial assays

To evaluate the response to antibiotics, semiquantitative *in vitro* susceptibility testing by the agar disk diffusion method was performed according to the manufacturer's recommendations (BBL, Becton, Dickinson and Company, Sparks, MD). The disc content was as followed: tetracycline (30 µg) and vancomycin (30 µg) (BD BBL, Franklin Lakes, NJ) and ampicillin (10 µg), trimethoprim (1.25 µg)/sulfamethoxazole (23.75 µg), gentamicin (120 µg), amoxicillin (20 µg)/clavulonic acid (10 µg), nalidixic acid (30 µg), cephalothin (30 µg) and enrofloxacin (5 µg) (Remel, Lenexa, KS). After 48 hours of incubation under microaerobic conditions, the sensitivity to each drug was quantified by measuring the zone of growth inhibition in millimeters. Because no standardized interpretive criteria exist for *Campylobacter* spp., the inhibition zone diameters were interpreted based on the interpretative criteria for the family *Enterobacteriaceae* [53]. Three independent experiments were performed in duplicates.

### *In vitro* cell line culture procedures

A stock culture of T84 (a human colonic epithelial cell line; ATCC, CCL 6) was maintained in 1∶1 mixture of Han's F12 and Dulbecco's modified Eagles medium (DMEM∶F12; ATCC) supplemented with 5% (vol/vol) fetal bovine serum, penicillin (100 U ml^−1^) and streptomycin (100 µg ml^−1^) according to ATCC's recommendation. RAW 264.7 murine macrophages (ATCC) were propagated in DMEM (ATCC) supplemented with 5% FCS (ATCC). The cell line cultures were maintained in 37°C humidified incubator aerated with 5% CO_2_. Culture media was replaced biweekly with new growth media to maintain the cell line.

### Cell adhesion and invasion (gentamicin-protection) assays in T84 and RAW 267.4 cells

*C. jejuni* was propagated on blood agar for 16 hours under microaerobic conditions before bacteria were collected and used in the experiment. Viable organisms were predominantly spirally shaped and no contamination by other types of bacteria was noted as visualized by Gram's staining. Cell adhesion and gentamicin-protection assays were performed according to the published protocol with some modifications [54]. Briefly, approximately 8×10^5^ of T84 and 2×10^5^ RAW 267.4 cells were initially seeded per well in a 12–well and 24-well tissue culture plate, respectively. T84 and RAW 267.4 were infected with *C. jejuni* with the MOI of 15 and MOI of 9, respectively. The cell monolayer was incubated with bacteria for 4 hours and was washed five times with 1× PBS to remove bacteria that failed to bind to the cells. The cell monolayer was treated with 1% saponin (Sigma) for 15 min and vortexed for 1 minute to release the bacteria. Enumeration of total adhered bacteria was performed by 10-fold serial dilution and plating.

Gentamicin-protection assays were performed according to the cell adhesion assay described above. However, after 4 hours of incubation, the culture was rinsed 3 times with 1× PBS and incubated for additional 2 hours in culture medium containing gentamicin (250 µg ml^−1^). *C. jejuni* was not recovered from the supernatant with this concentration of gentamicin and duration of treatment. The released intracellular bacteria were enumerated by serial dilutions and plating. Three independent assays were performed in triplicates or quadruplicates.

### Gene expression analysis

Total RNA was obtained from *C. jejuni* using a combination of Trizol and RNeasy Mini kit (Qiagen) according to the manufacturer's recommendations. For temporal gene expression analysis, bacteria were grown in biphasic growth medium with or without 0.1% DCA supplementation under microaerobic conditions. After 12, 24 and 48 hours, bacterial samples were collected by centrifugation and immediately suspended in 1 ml of Trizol (Invitrogen). The RNA was subjected to DNase treatment using the RNase-Free DNase set (Qiagen), purified with RNeasy Mini Kit, and quantified using Nanodrop 2000C spectrophotometer (Thermo Fischer Scientific, Barrington, IL). cDNA synthesis was performed using 200 ng of total RNA, 500 nM of each reverse primer (Table 3), and components of the High Capacity cDNA Reverse Transcription Kit (Applied Biosystem). The cDNA equivalent to 25 ng of total RNA for each of the genes was subjected to q-PCR with gene-specific primers (Table 3) using Fast SYBR Green Master Mix (AB Applied Biosystem, Carlsbad, CA). *cmeA* and *glyA* primers used for q-PCR were obtained from previously published studies [55]. Standard curves for the transcripts of each gene examined were created using a serial 10-fold dilution of 10^6^ to 10^1^ copies of the ATCC 43431 genome. Based on the known genome size of several *C. jejuni* strains deposited at the http://www.xbase.ac.uk/campydb/, the size of the *C. jejuni* ATCC 43431genome was estimated to be 1.6 megabases. The expression of all genes investigated was normalized to *glyA* previously shown to be unaffected by DCA [55]. No significant level of DNA contaminant was detected from all total RNA samples analyzed as determined by q-PCR. All samples were run in duplicates or triplicates in three independent experiments.

### Mouse strain and bacterial infection

B6.129P2-*IL-10^tm1Cgn^* mice originally from Jackson Laboratories (Bar Harbor, ME) were bred in house and maintained under barrier conditions in a facility accredited by the Association for Assessment and Accreditation of Laboratory Care International. Mice were free of known murine viruses, *Salmonella* spp., *Citrobacter rodentium*, ecto- and endoparasites, and known *Helicobacter* spp. Mice were housed in microisolater, solid-bottomed polycarbonate cages on hardwood bedding, fed a commercial pelleted diet, and administered water ad libitum. The mouse protocol was approved by the MIT Animal Care Committee. Thirty eight mice (19 males and 19 females) were divided into four experimental groups: Brucella sham control (4 males and 4 females), WT *C. jejuni* 43431 (5 males and 5 females), Δ*icmF1* (5 males and 5 females) and Δ*icmF2* (5 males and 5 females). Six to 7 week old mice were orally gavaged with either Brucella broth or approximately 10^8^ CFUs of *C. jejuni* WT, Δ*icmF1*, or Δ*icmF2* suspended in sterilized Brucella broth. All mice were euthanized by CO_2_ inhalation at 30 days p.i. and cecal tissues and fecal samples were aseptically collected for quantification of *C. jejuni* by q-PCR to evaluate for *C. jejuni* colonization.

### Real-time quantitative PCR for *C. jejuni* in cecum and feces of IL10-deficient mice

Cecal DNA was isolated using a Roche High Pure PCR template preparation kit. Fecal DNA was isolated using QIAamp DNA Stool Mini Kit (Qiagen). *C. jejuni* ATCC 43431 quantification was performed by real time q-PCR using *C. jejuni* 43431 *cytotoxin distending toxin* (*cdt*) *A*-specific primers (Table 3). The standard curve for *C. jejuni* 43431 *cdtA* was prepared as mentioned above. *cdtA* copy numbers of *C. jejuni* 43431 were expressed per microgram of murine chromosomal DNA and measured by q-PCR using a mammalian 18S rRNA gene-based primer and probe mixture (Applied Biosystems). The assay was run in duplicates.

### Statistical analysis

Statistical analysis was performed using student's t-test or Mann-Whitney U non-parametric test with p values≤0.05 considered as significant. All graphs were generated using GraphPad Prism 4.0 (GraphPad Software, Inc., LaJolla, CA).

## Supporting Information

Figure S1 **Confirmation of Δ** ***hcp*** **mutants, Δ** ***icmF*** **mutants, the complemented Δ** ***hcp1*** **strain, and the organization of** ***hcp*** **and** ***icmF.*** PCR amplification of genomic DNA from (A) WT *C. jejuni* ATCC 43431, isogenic Δ*hcp1*, Δ*hcp2*, Δ*icmF1*, Δ*icmF2* mutants harboring *cat* , the complemented Δ*hcp1* strain (Δ*hcp1*(*hcp*+)), the control *hcp1* mutant carrying *kat* (Δ*hcp1*(*kat*)), and (B) isogenic Δ*hcp3*, Δ*hcp4*, Δ*icmf3*, Δ*icmF4* mutants harboring *kat*, demonstrating successful replacement of the *hcp* and *icmF* WT alleles with mutant alleles interrupted by the *cat* (Δ*hcp1* and Δ*hcp2*: 1350 bp; Δ*icmF1* and Δ*icmF2*: 1407 bp) or *kat* (Δ*hcp3* and Δ*hcp4*:2079 bp; Δ*icmF3* and Δ*icmF4*: 2137 bp) as well as the complemented *P_CAT_::hcp::kat* and the control *P_CAT_::kat* into the *hcp1* mutant's 16S–23S rRNA spacer region. (C) Organization and orientation of *hcp* and *icmF* with respect to the neighboring genes in *C. jejuni* 43431. Opened triangles represent sites of insertion of *cat* and *kat*. (D) A schematic depiction of the constructs for complementation of Δ*hcp1.*
(TIF)

Figure S2 **Growth and mobility of WT** ***C. jejuni*** **, the Δ** ***hcp1*** **mutant, the Δ** ***icmF1*** **mutant, and the complemented Δ** ***hcp1*** **strain, Δ** ***hcp1*** **(** ***hcp*** **+).** (A) Growth dynamics of WT, Δ*hcp* and Δ*icmF* mutants, the complemented Δ*hcp1* strain, and the Δ*hcp1* mutant containing only *kat* integrated in the 16S–23S spacer region. (B) The swarming ability of WT, the Δ*hcp* mutant, the Δ*icmF* mutant, and the complemented Δ*hcp1* strain. The results presented are representative of three independent experiments.
(TIF)

Figure S3 **Growth dynamics of WT** ***C. jejuni*** **, the Δ** ***hcp1*** **mutant, and the Δ** ***icmF1*** **mutant in the physiologically relevant concentrations of DCA.** (A) Ten-fold serial dilutions of overnight cultures were spotted on the agar plates containing 0% to 0.05% DCA and growth was evaluated at 12, 18, 36 and 48 hours. (B) Ten-fold serial dilutions were performed and CFUs of WT and Δ*hcp1* mutant were determined at various time points during growth in liquid media lacking or supplemented with 0.1% DCA and 0.2% DCA. The results are representative of three independent experiments. Error bars represent the standard error of the mean. The data points overlap at multiple time points for WT and the Δ*hcp1* mutant. P value: *≤0.05, **≤0.01.
(TIF)

Figure S4 **Expression levels of** ***hcp*** **in WT** ***C. jejuni*** **, the Δ** ***hcp1*** **mutant, the Δ** ***icmF1*** **mutant, and the complemented Δ** ***hcp1*** **strain, Δ** ***hcp1*** **(** ***hcp*** **+), grown for 14 hours in liquid media.** The results are representative of three independent experiments. Error bars represent the standard error of the mean. P value: ***≤0.001.
(TIF)
