# Supplementary figures and images for: Campylobacter jejuni Type VI Secretion System: Roles in Adaptation to Deoxycholic Acid, Host Cell Adherence, Invasion, and In Vivo Colonization
Source: PLoS One. 2012 Aug 27;7(8):e42842. doi: 10.1371/journal.pone.0042842 (PMC3428339; doi:10.1371/journal.pone.0042842)

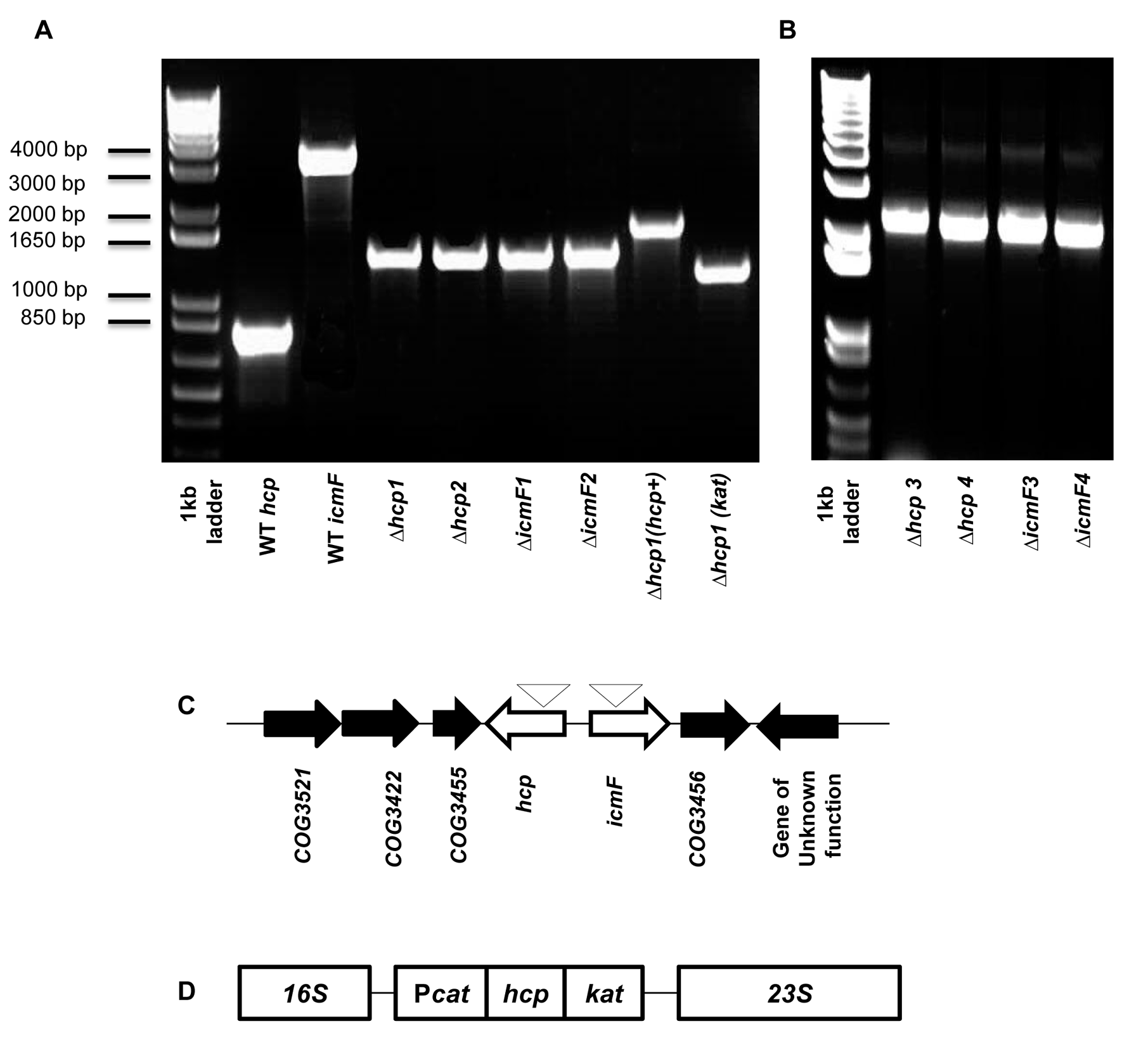

Supplement: Figure S1 — Confirmation of Δ hcp mutants, Δ icmF mutants, the complemented Δ hcp1 strain, and the organization of hcp and icmF. PCR amplification of genomic DNA from (A) WT C. jejuni ATCC 43431, isogenic Δhcp1, Δhcp2, ΔicmF1, ΔicmF2 mutants harboring cat , the complemented Δhcp1 strain (Δhcp1(hcp+)), the control hcp1 mutant carrying kat (Δhcp1(kat)), and (B) isogenic Δhcp3, Δhcp4, Δicmf3, ΔicmF4 mutants harboring kat, demonstrating successful replacement of the hcp and icmF WT alleles with mutant alleles interrupted by the cat (Δhcp1 and Δhcp2: 1350 bp; ΔicmF1 and ΔicmF2: 1407 bp) or kat (Δhcp3 and Δhcp4:2079 bp; ΔicmF3 and ΔicmF4: 2137 bp) as well as the complemented PCAT::hcp::kat and the control PCAT::kat into the hcp1 mutant's 16S–23S rRNA spacer region. (C) Organization and orientation of hcp and icmF with respect to the neighboring genes in C. jejuni 43431. Opened triangles represent sites of insertion of cat and kat. (D) A schematic depiction of the constructs for complementation of Δhcp1. (TIF) [file pone.0042842.s001.tif]

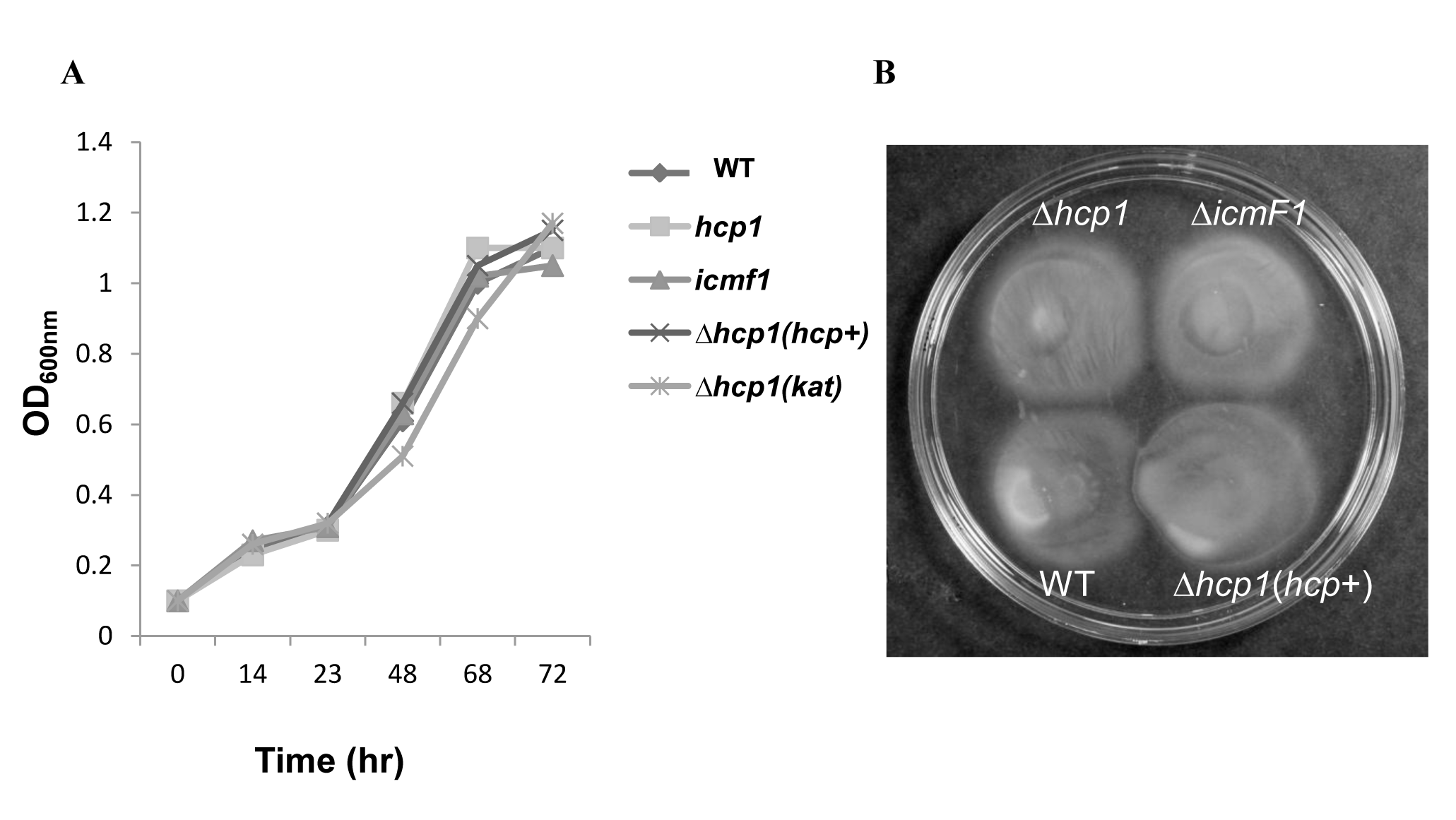

Supplement: Figure S2 — Growth and mobility of WT C. jejuni , the Δ hcp1 mutant, the Δ icmF1 mutant, and the complemented Δ hcp1 strain, Δ hcp1 ( hcp +). (A) Growth dynamics of WT, Δhcp and ΔicmF mutants, the complemented Δhcp1 strain, and the Δhcp1 mutant containing only kat integrated in the 16S–23S spacer region. (B) The swarming ability of WT, the Δhcp mutant, the ΔicmF mutant, and the complemented Δhcp1 strain. The results presented are representative of three independent experiments. (TIF) [file pone.0042842.s002.tif]

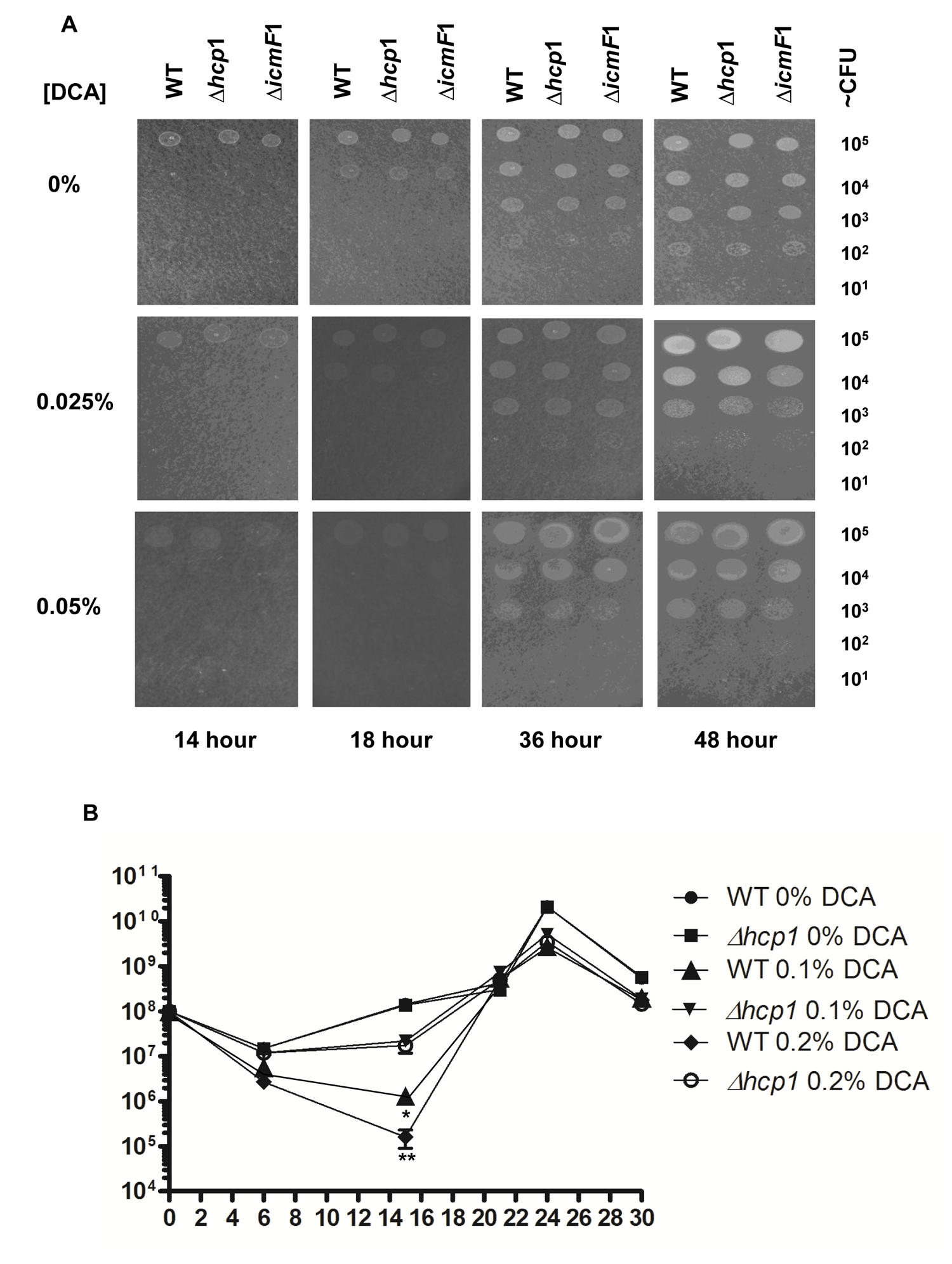

Supplement: Figure S3 — Growth dynamics of WT C. jejuni , the Δ hcp1 mutant, and the Δ icmF1 mutant in the physiologically relevant concentrations of DCA. (A) Ten-fold serial dilutions of overnight cultures were spotted on the agar plates containing 0% to 0.05% DCA and growth was evaluated at 12, 18, 36 and 48 hours. (B) Ten-fold serial dilutions were performed and CFUs of WT and Δhcp1 mutant were determined at various time points during growth in liquid media lacking or supplemented with 0.1% DCA and 0.2% DCA. The results are representative of three independent experiments. Error bars represent the standard error of the mean. The data points overlap at multiple time points for WT and the Δhcp1 mutant. P value: *≤0.05, **≤0.01. (TIF) [file pone.0042842.s003.tif]

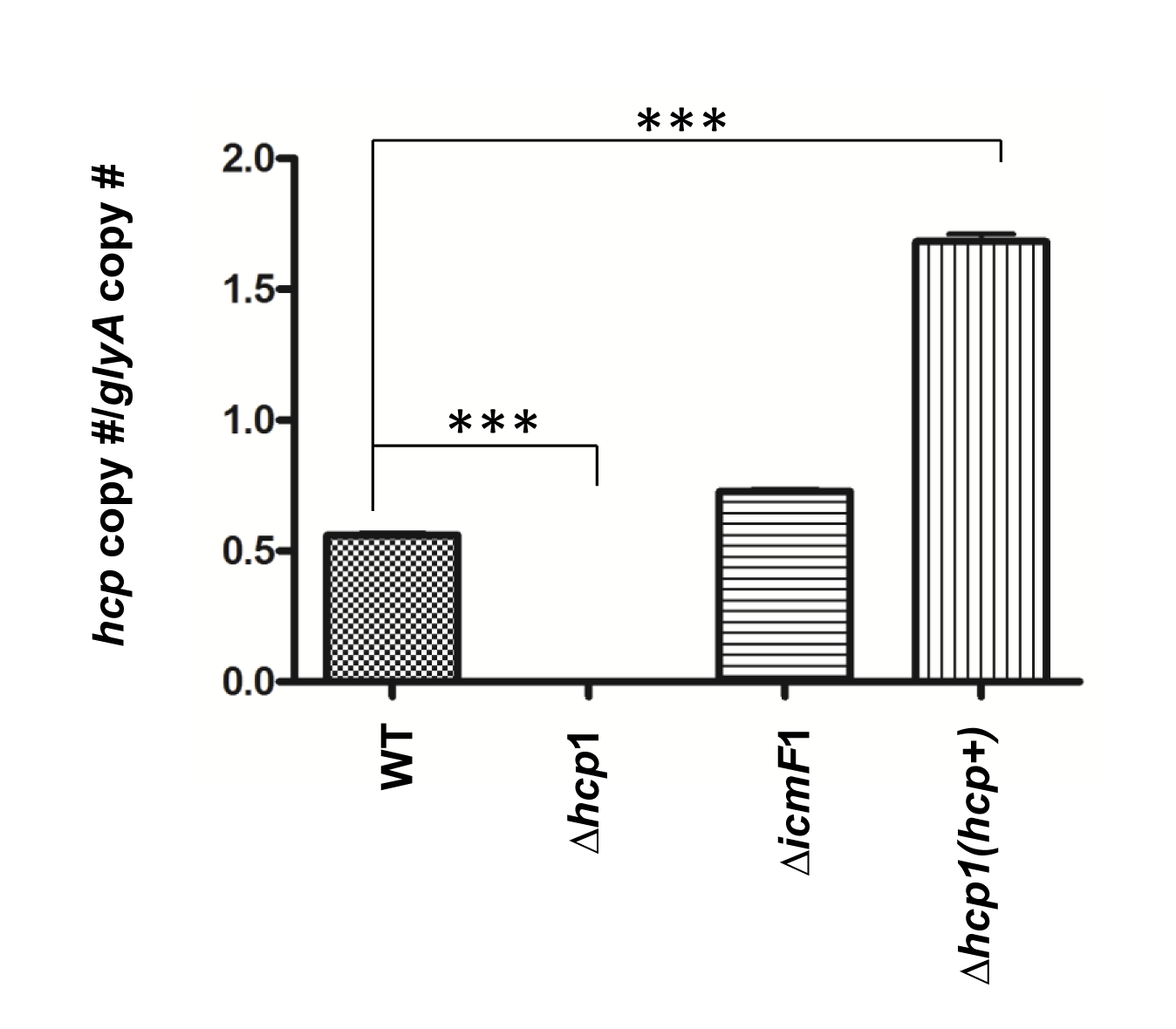

Supplement: Figure S4 — Expression levels of hcp in WT C. jejuni , the Δ hcp1 mutant, the Δ icmF1 mutant, and the complemented Δ hcp1 strain, Δ hcp1 ( hcp +), grown for 14 hours in liquid media. The results are representative of three independent experiments. Error bars represent the standard error of the mean. P value: ***≤0.001. (TIF) [file pone.0042842.s004.tif]
